# UPP1 enhances bladder cancer progression and gemcitabine resistance through AKT

**DOI:** 10.7150/ijbs.83774

**Published:** 2024-01-27

**Authors:** Wenzhi Du, Sheng Tu, Wenxiu Zhang, Yi Zhang, Wei Liu, Kangping Xiong, Fenfang Zhou, Na Li, Renjie Zhang, Jingtian Yu, Mingxing Li, Wan Xiang, Kaiyu Qian, Gang Wang, Yu Xiao, Xinghuan Wang, Lingao Ju

**Affiliations:** 1 Hubei Key Laboratory of Urological Diseases, Laboratory of Precision Medicine, Zhongnan Hospital of Wuhan University, Wuhan, China.; 2 Department of Urology, The First Affiliated Hospital of Shandong First Medical University & Shandong Provincial Qianfoshan Hospital, Shandong Medicine and Health Key Laboratory of Organ Transplantation and Nephrosis, Shandong Institute of Nephrology, Jinan, Shandong, China.; 3 Department of Urology, Zhongnan Hospital of Wuhan University, Wuhan, China.; 4 Department of Pediatrics, Maternal and Child Health Care Hospital of Shandong Province, Jinan, China.; 5 Euler Technology, ZGC Life Sciences Park, Beijing, China.; 6 Center for Quantitative Biology, School of Life Sciences, Peking University, Beijing, China.; 7 Department of Urology, Aerospace Center Hospital, Peking University Aerospace School of Clinical Medicine, Beijing, China.; 8 Department of Radiology, Zhongnan Hospital of Wuhan University, Wuhan, China.; 9 State Key Laboratory of Ophthalmology, Zhongshan Ophthalmic Center, SunYat-sen University, Guangzhou, China.; 10 Department of Biological Repositories, Human Genetic Resources Preservation Center of Hubei Province, Zhongnan Hospital of Wuhan University, Wuhan, China.; 11 Medical Research Institute, Frontier Science Center of Immunology and Metabolism, TaiKang Center for Life and Medical Sciences, Wuhan University, Wuhan, China.

**Keywords:** UPP1, AKT, gemcitabine, metastasis, bladder cancer.

## Abstract

UPP1, a crucial pyrimidine metabolism-related enzyme, catalyzes the reversible phosphorylation of uridine to uracil and ribose-1-phosphate. However, the effects of UPP1 in bladder cancer (BLCA) have not been elucidated. AKT, which is activated mainly through dual phosphorylation (Thr308 and Ser473), promotes tumorigenesis by phosphorylating downstream substrates. This study demonstrated that UPP1 promotes BLCA cell proliferation, migration, invasion, and gemcitabine resistance by activating the AKT signaling pathway *in vitro* and *in vivo*. Additionally, UPP1 promoted AKT activation by facilitating the binding of AKT to PDK1 and PDK2 and the recruitment of phosphatidylinositol 3,4,5-triphosphate to AKT. Moreover, the beneficial effects of UPP1 on BLCA tumorigenesis were mitigated upon UPP1 mutation with Arg94 or MK2206 treatment (AKT-specific inhibitor). AKT overexpression or SC79 (AKT-specific activator) treatment restored tumor malignancy and drug resistance. Thus, this study revealed that UPP1 is a crucial oncogene and a potential therapeutic target for BLCA and that UPP1 activates the AKT signaling pathway and enhances tumorigenesis and drug resistance to gemcitabine.

## Introduction

According to GLOBOCAN epidemiological data from 2020, there were 573,000 newly diagnosed bladder cancer (BLCA) cases and 213,000 deaths related to BLCA worldwide, which poses a severe threat to human health 1. Based on the depth of muscle invasion, BLCA can be mainly classified into non-muscle-invasive BLCA (NMIBC) and muscle-invasive BLCA (MIBC) 2. MIBC accounts for almost 70% of organ-confined BLCA 3, while 15%-20% of NMIBC tumors are transformed into MIBC, which is associated with an increased mortality rate 4. Approximately 50% of patients with MIBC develop metastasis before undergoing radical surgery 5. The major limiting factor for BLCA treatment is the high recurrence rate 6. The main treatment options for BLCA include transurethral resection of bladder tumors, laparoscopic radical cystectomy, and adjuvant chemotherapy (e.g., gemcitabine) 7, 8. However, these therapeutic strategies have not markedly improved the overall survival (OS) rate of patients with BLCA 9. Researchers have identified the genes and pathways that drive the pathogenesis of urothelial carcinoma through genome-wide expression and sequencing studies. More than one molecular subclass of urothelial carcinoma has been identified, spanning traditional rank and stage categories 10. The molecular mechanisms underlying BLCA tumorigenesis must be elucidated to identify novel targeted therapies and enhance BLCA patients' quality of life and prognosis 11.

AKT, a serine/threonine kinase, mediates several signal transduction pathways 12. Additionally, AKT regulates downstream proteins that adjust cell proliferation, migration, and apoptosis, including FOXO1 and GSK3β. The expression of AKT is dysregulated in various malignancies 13. AKT activity is upregulated in BLCA, promoting tumor cell proliferation and inducing metabolic alterations 14. Various upstream growth signals activate AKT. Two critical activating sites have been identified in AKT (Thr308 and Ser473) 15. Activated phosphoinositide 3-kinase (PI3K) is reported to promote phosphatidylinositol 3,4,5-triphosphate (PIP_3_) generation and help AKT activation in the cell membrane 16. Phosphatase and tensin homolog (PTEN) antagonizes PI3K by dephosphorylating PIP_3_ to generate phosphatidylinositol 4,5-bisphosphate (PIP_2_), inhibiting AKT activation 17. AKT binds to PIP_3_ through its pleiotropic substrate protein homology (PH) domain, promoting the translocation of AKT to the cell membrane where its Thr308 site is phosphorylated by PDK1 18 and its Ser473 site is phosphorylated by PDK2 19, leading to classic activation. Various clinical trials have reported that suppression of the AKT signaling pathway is associated with tumor regression 20. The Food and Drug Administration approved several inhibitors of the AKT signaling pathway based on their efficacy and safety in clinical trials 21. Thus, treatments that target AKT can be integrated into cancer prevention and management.

Tumor development is critically dependent on pyrimidine metabolism. Several drugs targeting key pyrimidine metabolism-related enzymes have been applied in clinical practice 22, 23. Uridine phosphorylase, a crucial enzyme for maintaining uridine homeostasis and salvaging pyrimidines, catalyzes the reversible phosphorylation of uridine to uracil and ribose-1-phosphate 24. Previous studies have identified UPP1 and UPP2 as two types of uridine phosphorylases. The distribution and expression of UPP1 are higher than those of UPP2 25. *UPP1* expression is associated with the outcome of different types of malignancies 26, 27. However, it remains unclear whether UPP1 contributes to BLCA tumorigenesis.

This study demonstrated that UPP1 was upregulated in human BLCA tissues and elucidated the consequences of UPP1 upregulation in different BLCA cell lines *in vitro* and *in vivo*. Additionally, UPP1 was demonstrated to interact with the C-terminus of AKT and upregulate the activity of AKT. UPP1 stimulates cell proliferation, migration, invasion, and gemcitabine resistance and inhibits apoptosis and reactive oxygen species (ROS) generation in BLCA through the AKT signaling pathway. In contrast, the UPP1 mutant UPP1-R94A did not interact with AKT. The functional role of UPP1 in human BLCA pathogenesis demonstrated in this study provides novel ideas for diagnosing and treating BLCA.

## Materials and Methods

### Cell culture and reagents

SV-HUC-1 (human uroepithelial cells), HEK-293T (human embryonic kidney cells), UM-UC-3, J82, SCaBER, 5637, T24, and RT4 (human BLCA cell lines) were supplied by the Cell Bank of Chinese Academy of Science (Shanghai, China) with authentication. According to the recommended cell culture protocols, Roswell Park Memorial Institute-1640 medium, minimal essential medium, Dulbecco's modified Eagle's medium, and McCoy's 5A medium (Gibco, USA) were used to culture the above cell lines with 10% fetal bovine serum (FBS) and 1% penicillin-streptomycin solution (Gibco, USA). Cells were cultured at 5% CO_2_ and 37℃. Supplementary Table S1 contains the catalog numbers and commercial sources of the antibodies utilized in this research. The AKT-specific inhibitor MK2206 (S1078, Selleck), the AKT-specific activator SC79 (HY-18749, MedChemExpress), and gemcitabine (S1149, Selleck) were obtained from the indicated commercial sources.

### Short-interfering RNAs (siRNAs) and plasmid construction and transfection

Shanghai GenePharma Co., Ltd. provided the siRNAs against UPP1 (siUPP1). Supplementary Table S2 contains the listings of siUPP1 sequences. The UPP1 overexpression plasmid (Cat. #EX-Q0192-M35) was purchased from OmicsLink^TM^. The plasmid encoding UPP1-R94A was constructed based on wild-type (WT) UPP1 (UPP1-WT). Supplementary Table S3 contains the primer sequences for UPP1-R94A plasmid construction. All plasmids were subjected to sequencing before transfection to ensure that they were mutation-free. Lipofectamine 3000 reagent (Invitrogen, USA) was used for transfection.

### RNA extraction, reverse transcription, and quantitative reverse transcription PCR (qRT-PCR)

HiPure total RNA mini kit (Cat. #R4111, Magen, China) was used to extract total RNA, following the product's instructions. The isolated RNA (1 μg) was reverse-transcribed into complementary DNA (cDNA). iQ^TM^ SYBR^®^ Green Supermix (Bio-Rad, USA) and cDNA (500 ng) were included in 20 μL qRT-PCR reaction volume. Supplementary Table S4 contains the primer sequences for *UPP1* and *ACTB*. The expression levels of *ACTB* were used to normalize those of target genes.

### Methyl thiazolyl tetrazolium (MTT) assay

Pretreated cells were seeded in five repetitive 96-well plates for five days (3000 cells/well). The cells in each well were cultured in 200 μL medium with 10% FBS. After one day of incubation, the cells of the first 96-well plate were incubated with 20 μL of MTT (Sigma) for four hours. After discarding the supernatant, the cells were solubilized in 150 μL of dimethyl sulfoxide (DMSO). The spectrophotometer (Spectramax M5, Molecular Devices) was used to examine the reaction mixture's absorbance (570 nm). The remaining 96-well plates were individually subjected to the MTT assay at the same time each day for the next four days.

### Colony formation assay

Post-transfection cells (1000 cells/well) were seeded in a 6-well plate and cultured in an incubator for approximately nine days to form visible colonies. After being treated with 4% formaldehyde, the colonies were stained with a 0.1% solution of crystal violet for 30 mins. ImageJ software was used to count colonies.

### Flow cytometric analysis

Pretreated cells were washed thrice with cold phosphate-buffered saline (PBS, pH 7.4) for cell cycle analysis. Cells were then resuspended in 1× DNA staining solution, permeabilized with permeabilization solution, and stained with propidium iodide (Cat. #CCS012, Multisciences, China) in the dark at 37°C for 30 mins. To analyze the apoptosis rate, we harvested and incubated cells with the reagents of the annexin V-fluorescein isothiocyanate apoptosis analysis kit, following the manufacturer's instructions (Cat. #AO2001-02P-G, Sungene Biotech, China). To measure the intracellular ROS levels in BLCA cells, we harvested and washed post-transfection cells with cold PBS. Next, the cells were resuspended in 1 mL FBS-free medium and incubated with 10 mM 2',7'-dichlorofluorescein diacetate (a fluorescent probe) for 30 mins in the dark at 37℃ (Cat. #D6883, Sigma-Aldrich, USA). Cells were then thoroughly washed thrice to remove the unbound probe and analyzed using a flow cytometer (Cat. #FC500, CytoFLEX S, Beckman, USA). The raw data were processed using FlowJo software (v10.8.1).

### Transwell assay

To perform the migration assay, we filled 24-well plates with polycarbonate transwell filters (8-μm pore size, Corning, USA). Next, 200 μL of FBS-free medium inoculated with cells (4 × 10^4^ UM-UC-3 and T24 cells; 1 × 10^5^ cells for SCaBER and 5637 cells) was added to the upper chamber. FBS-containing medium was placed in the lower chamber (600 μL). After 24 hrs of incubation, the cells were fixed with 4% paraformaldehyde and stained with 0.1% crystal violet for 30 mins. Cotton swabs were used to wipe the cells from the inner layers of the filter, and the chamber was carefully washed and dried. The migrated cells were imaged under an inverted microscope. ImageJ software was used to calculate the number of migrated cells. Matrigel was added to the chamber to perform the invasion assay, and the chamber was incubated for 1 hr at 37℃ to cover the filter. The other protocols for the invasion assay were identical to those for the migration assay.

### Wound healing assay

Equivalent transfected cells were placed in a 6-well plate at optimal confluency (80-90%). After 48 hrs, when the cells reached 100% confluency, the cell monolayer was scratched with a pipette tip. The image of the monolayer at 0 hrs was captured. After being washed with PBS, the cells were cultured in FBS-free medium for 36 hrs. The image of the monolayer was captured at 36 hrs. The wound healing percentages at 0 and 36 hrs were compared (100% - (scratch area at 36 hrs/scratch area at 0 hrs)) in different groups.

### Immunoblotting analysis

Cells were washed with PBS and lysed on ice for 30 mins with radioimmunoprecipitation assay lysis buffer containing protease and phosphatase inhibitors. Next, the lysate was centrifuged at 3000 rpm and 4℃ for 10 mins. The bicinchoninic acid (BCA) assay kit (Cat. #P0011, Beyotime, China) was utilized to assess the protein concentration in the supernatant. Denaturation of the samples was carried out using sodium dodecyl sulfate (SDS) buffer at 95 ℃ for 10 mins. SDS-polyacrylamide gel electrophoresis was performed on the protein samples. The gel percentage depends on the molecular weight of the proteins to be tested. The resolved proteins were transferred to a polyvinylidene fluoride membrane (0.45-μm pore size). To prevent nonspecific binding, we blocked the membrane with 5% skim milk in 1× Tris-buffered saline containing Tween-20 (20 mM Tris, 150 mM NaCl, 0.1% Tween-20, pH 7.4) for 2 hrs at room temperature (RT, 18-25 °C). Next, the indicated primary and secondary antibodies were probed onto the membrane. Immunoreactive signals were developed using chemiluminescence and imaged using a gel imager (BioSpectrum 515 Imaging System, UVP).

### Co-immunoprecipitation (co-IP) analysis

A lysis buffer (150 mM NaCl, 150 mM Tris, 0.4% NP-40, pH 7.4) containing protease and phosphatase inhibitors was used to lyse cells. The lysate was incubated with 1 μL of undiluted target antibody at 4 ℃ for 4-6 hrs to ensure the binding of the target protein to the antibody. Next, protein A magnetic beads were washed thrice with lysis buffer. Each sample was incubated with 20 μL of beads at 4°C for 2 hrs to ensure effective binding between antibodies and beads. The supernatant was removed, and the beads were washed thrice with lysis buffer. The protein-antibody-bead complex was eluted with 1× SDS buffer and boiled at 100 °C for 10 mins. Immunoblotting was performed to test the expression of the indicated proteins.

### Glutathione S transferase (GST) pull-down assay

His-UPP1 (Cat. #ab101152, Abcam, UK), GST-AKT (Cat. #CSB-EP001553HU, CUSABIO, China), and GST (Cat. #CSB-RP101744Ba, CUSABIO, China) fusion proteins were obtained from the indicated commercial sources. GST and GST-AKT fusion proteins (2 μg) were dissolved in buffer (150 mM NaCl, 150 mM Tris, 0.4% NP-40, pH 7.4) with protease and phosphatase inhibitors and incubated with 20 μL of washed glutathione-Sepharose beads at 4 °C for 2 hrs. The beads were washed thrice with washing buffer and incubated with 2 μg His-UPP1 fusion protein at 4 ℃ for 2 hrs. The fused protein-GST protein-bead complex was eluted with 1× SDS buffer and denatured for 10 mins at 100 °C. The expression levels of the indicated proteins in each sample were evaluated by immunoblotting analysis.

### AKT kinase activity assay

The AKT enzyme activity in the cellular extract was analyzed with the AKT kinase activity kit (Cat. #ADI-EKS-400A, Enzo, USA), following the procedures of the product manual. The protein concentration in the sample was examined using the BCA protein assay kit (Cat. #P0011, Beyotime, China). The sample volume for the AKT kinase activity assay was 30 μL. The absorbance of the reaction mixture at 450 nm was measured to determine AKT kinase activity. Absorbance (450 nm)/protein mass (mg) was calculated to obtain the quantified AKT kinase activity.

### PIP_3_ pull-down assay

The PIP_3_ beads (Cat. #P-B00S, Echelon Biosciences, USA) were centrifuged at 800 *g* and resuspended in binding buffer (150 mM NaCl, 150 mM Tris, 0.4% NP-40, pH 7.4). Subsequently, the cells were lysed with binding buffer. Each sample was incubated at 4 °C with 100 μL PIP_3_ beads for 3 hrs. The solution containing protein and beads underwent 4 washes with washing buffer. The bound proteins were eluted with 1× SDS loading buffer and denatured at 100 °C for 10 mins. The complex was analyzed using immunoblotting analysis.

### Immunofluorescence

The transfected cells were pre-seeded on slides, fixed with 4% formaldehyde for 30 mins, and incubated with a buffer solution (2% bovine serum albumin and 0.3% Triton X-100) for 40 mins. Next, the cells were washed thrice with PBS and sequentially incubated with the primary antibodies (4 ℃, overnight), secondary antibodies (RT, 2 hrs), and 4',6-diamidino-2-phenylindole (1:1000, RT, 5 mins). The samples were sealed, air-dried, and imaged under a confocal microscope (Nikon, Japan).

### Hematoxylin and eosin (H&E) staining and immunohistochemical (IHC) analysis

The paraffin sections (5 mm) of xenograft tissue samples were subjected to H&E staining. The dewaxed sections were cleared with xylene and rehydrated in an ethanol series (100%, 96%, 80%, 70%, and H_2_O) at the indicated time points and stained with 10% hematoxylin (Sigma-Aldrich, USA). The sample was washed and stained with a solution of 1% eosin (Sigma-Aldrich, USA) containing 0.2% glacial acetic acid. After each step, the sections were immediately cleared with xylene and dehydrated in an ethanol series (70%, 80%, 96%, and 100%). An inverted phase contrast microscope was used to image the sections. To perform IHC analysis, the tissue samples of experimental animals and tissue microarray slides were sequentially formalin-fixed, paraffin-embedded, sectioned, deparaffinized, and hydrated. Citrate buffer (pH 6.0) was used for antigen retrieval. The sections were incubated with H_2_O_2_ (0.3%) to block endogenous peroxidase activity and sequentially probed with primary and secondary antibodies. Immunoreactive signals were developed using peroxidase and 3,3'-diaminobenzidine (DAB) tetrahydrochloride reactions. Finally, the samples were incubated in DAB (5-10 mins).

### Xenograft mouse model

Shanghai GenePharma Co., Ltd. provided the lentivirus packaged with short hairpin RNA against *UPP1* (shUPP1) or negative control shRNA (shNC); Supplementary Table S2 lists the sequences. UM-UC-3 cells were infected with lentivirus. The recombinant cells were screened using 1 μg/mL puromycin (Sigma, USA). Wuhan BNT Bioscience Co., Ltd. provided male BALB/c nude mice aged 6 weeks. Mice were randomly assigned to different groups. To examine the function of UPP1 in BLCA cell proliferation *in vivo*, we randomly established two groups (n = 5) of nude mice. Mice were subcutaneously injected with shNC-transfected or shUPP1-transfected UM-UC-3 cells (1 × 10^6^ cells) diluted in 100 μL cold PBS (for each point) at two different points. The volume of the tumor was regularly determined using a vernier caliper. The tumor was allowed to grow for 25 days. The tumors were harvested via surgery from the experimental animals for subsequent experiments. To investigate metastasis *in vivo*, we randomly divided mice into two groups (n = 4). shNC-transfected or shUPP1-transfected UM-UC-3 cells were intravenously injected (1 × 10^6^ cells) via the tail vein. After six weeks of tumor cell growth, the fluorescence intensity of green fluorescent protein (GFP)-expressing cells was determined using Living Image software (Caliper Life Sciences). The lung tissue was excised and subjected to H&E staining. To evaluate *in vivo* tumor gemcitabine resistance, we randomly divided mice into four groups (n = 4) and subcutaneously injected mice with shNC-transfected or shUPP1-transfected UM-UC-3 cells (1 × 10^6^ cells). The tumor was allowed to grow for 15 days until the tumors were visible. Next, the mice were intraperitoneally administered 200 μL of DMSO or gemcitabine (50 mg/kg bodyweight) twice a week according to the experimental groupings. Group allocation was blinded during experimental result evaluation. For the xenograft mouse model of UPP1 overexpression, T24 cells were transfected with lentiviruses containing UPP1 overexpression sequences and vector sequences (LV5) provided by Shanghai GenePharma Co., Ltd. As described above, the subcutaneous tumor mouse and the experimental lung metastasis mouse models were constructed. The IVIS Spectrum living imaging system was used to calculate the fluorescence intensity of GFP-expressing cells. Radiant efficiency was calculated by (p/sec/cm^2^/sr)/(μW/cm^2^). The color scale is annotated beside each figure. The Wuhan University Institutional Animal Care and Use Committee approved all experimental protocols (approval No. ZN2022030).

### Validation of diagnostic value from public databases and tissue microarrays

The Cancer Genome Atlas (TCGA)-BLCA RNA sequencing with clinical data was obtained from the TCGA database (*https://portal.gdc.com*). The GSE13507, GSE32894, and GSE48075 datasets were obtained from the Gene Expression Omnibus database (*https://www.ncbi.nlm.nih.gov/geo/*). Kaplan-Meier survival curves were generated using R (version 3.5.2) and R Bioconductor. The clinical value of UPP1 for diagnosing BLCA was demonstrated using receiver operating characteristic (ROC) curves 28. Shanghai Outdo Biotech provided the tissue microarray (Cat. #HBlaU079Su01). The slide was scanned entirely using an Apero VERSA 8 phase contrast microscope (Leica, German). Image-Pro Plus 6.0 was used to analyze the average optical density (AOD) to represent *UPP1* expression. All picture backgrounds were flattened with a max feature size of 840 (pix). Area and IOD were selected as measurements. To calibrate the optical density, we set the incident level at 255. The color segmentation was based on histogram hue-saturation-intensity (HSI) mode (H: 0-30, S: 0-254, I: 0-220). After applying the color filter, transparent white was selected, and the picture was turned to grayscale (8-bit). The target area's integral optical density (IOD) value was also automatically calculated. The AOD values are calculated as follows: AOD = IOD/area. Two pathologists checked the data accuracy.

### Gene set enrichment analysis (GSEA) and gene set variation analysis (GSVA)

Based on normalized TCGA-BLCA data, the median expression level of *UPP1* was chosen as the cutoff point for dividing the samples into high-expression and low-expression groups. Visualization of the analysis was performed using javaGSEA. The HALLMARK gene sets were selected as a reference, and the false discovery rate was calculated. The cutoff for determining significantly altered pathways was *p* < 0.05 29. To perform GSVA, TCGA datasets were analyzed using the R package GSVA with the method = 'ssgsea' as the parameter. Spearman correlation analysis was used to determine the correlation coefficients between genes and pathways. *p* < 0.05 was considered significant for differences.

### Statistical analysis

Mean ± standard deviation was used to represent all data. The experiments were repeated thrice each, and data from three individual experiments were represented in all analyses. Means between the different groups were compared using two-tailed Student's t-test and one-way analysis of variance followed by Tukey's post-hoc test. Kaplan-Meier analysis was performed to estimate the OS. Survival curves were compared using log-rank tests. SPSS v. 25.0. was used to perform all statistical analyses. *p* < 0.05 indicates significant difference in data. Levels of statistical significance are indicated as follows: *: *p* < 0.05; **: *p* < 0.01; ***: *p* < 0.001; ns: not significant (*p* > 0.05).

## Results

### Prognostic value of UPP1 in BLCA

**Figure 1:**
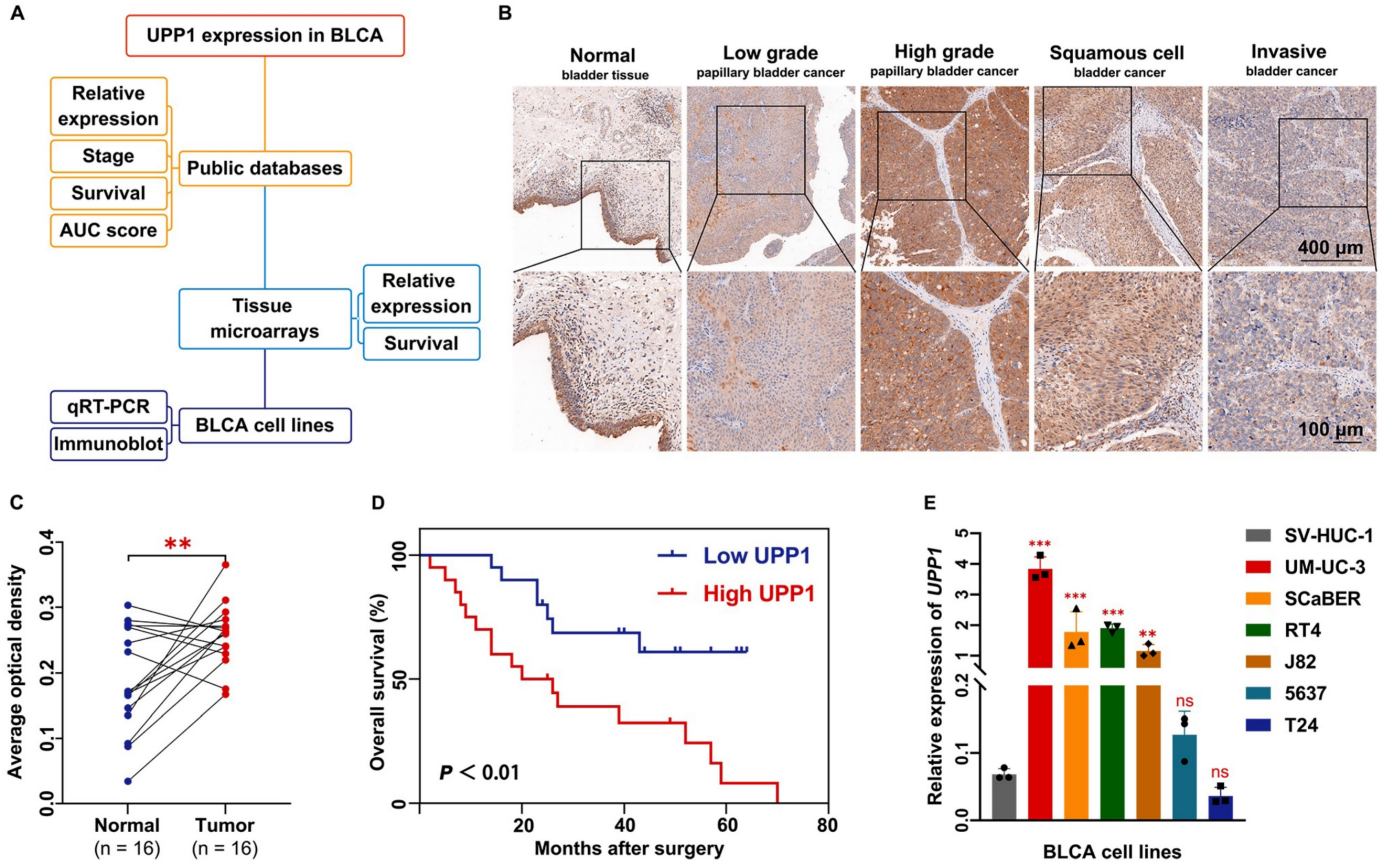
** UPP1 expression in BLCA. (A)** Diagram of evidence sources of UPP1 as an essential prognostic biomarker.** (B)** Immunohistochemical staining of UPP1 in different stages of BLCA from the tissue microarray. **(C)** Relative expression in paired adjacent tissues and BLCA tissues from the tissue microarray. **(D)** Kaplan-Meier analysis of overall survival of samples in the tissue microarray. **(E)** The relative expression of *UPP1* in BLCA cell lines was examined using qRT-PCR. *: *p* < 0.05; **: *p* < 0.01; ***: *p* < 0.001; ns: not significant (*p* > 0.05).

The *UPP1* expression levels in healthy and BLCA tissues curated in public databases and a tissue chip were investigated (Fig. 1A). RNA sequencing and clinical data were retrieved from the TCGA-BLCA dataset. Bioinformatics analysis of unpaired samples revealed that *UPP1* expression in tumor tissues was significantly higher than that in non-tumor tissues (Supplementary Fig. S1A). Furthermore, the results of paired sample analysis were consistent with those of unpaired sample analysis (Supplementary Fig. S1B). *UPP1* expression in the T3 and T4 stage tumors was upregulated when compared with that in the T1 and T2 stage tumors (Supplementary Fig. S1C). Next, data were mined from the Gene Expression Profiling Interactive Analysis (GEPIA) website (*http://gepia2.cancer-pku.cn*), which enables visualization and interactive analysis of cancer gene expression profiles based on TCGA and Genotype-Tissue Expression databases 30. GEPIA revealed that *UPP1* expression was positively correlated with BLCA stage (Supplementary Fig. S1D).

Additionally, a BLCA tissue chip was analyzed to further support the findings. The tissue chip was subjected to IHC. The AOD of each site was measured as an indirect indicator of gene expression 31, 32. The median value of AOD was selected as the cutoff point to classify the samples into high-expression and low-expression groups. The IHC staining intensity in BLCA tissues was higher than that in non-tumor tissues (Supplementary Fig. S1E). High-grade, squamous cell, and infiltrating BLCA tissue exhibited increased IHC staining intensity (Fig. 1B). Analysis of the AODs of paired (Fig. 1C) and unpaired (Supplementary Fig. S1F) samples revealed that *UPP1* expression in tumor tissues was higher than that in non-tumor tissues. To examine the clinical value of UPP1, ROC curves based on TCGA data were analyzed to evaluate the ability of UPP1 to differentiate between non-tumor and malignant tissues. The area under the curve value of UPP1 was 0.706 (Supplementary Fig. S1G), indicating good diagnostic accuracy.

Furthermore, data from the tissue microarray (Fig. 1D) and GSE13507 dataset (Supplementary Fig. S1H) were subjected to Kaplan-Meier survival analysis. The survival curve of the GSE13507 dataset was generated using the PrognoScan database (*http://dna00.bio.kyutech.ac.jp/PrognoScan/index.html*). UPP1 upregulation was associated with poor prognosis. Additionally, UPP1 expression was not correlated with other basic clinical parameters (gender and age) (Supplementary Table S5).

### UPP1 expression is positively correlated with BLCA cell proliferation, metastasis, and invasion *in vitro* and *in vivo*

The expression levels of UPP1 in multiple BLCA cell lines were examined at the transcriptional and translational levels. The expression of *UPP1* in UM-UC-3, SCaBER, RT4, and J82 cell lines was higher than that in SV-HUC-1 but was not significantly different in 5637 and T24 cell lines (Fig. 1E). The UPP1 protein levels in UM-UC-3, SCaBER, RT4, and J82 cell lines were higher than those in SV-HUC-1 but were lower in 5637 and T24 cell lines (Supplementary Fig. S1I). The knockdown efficiency and specificity of the three siRNAs were verified using qRT-PCR (Supplementary Fig. S2A-B) and immunoblotting analyses (Supplementary Fig. S2D). All three siRNA sequences were included in subsequent studies. The UPP1 overexpression plasmid was used to transfect cells. The overexpression efficiency of the plasmid was examined using qRT-PCR (Supplementary Fig. S2C) and immunoblotting analyses (Supplementary Fig. S2D).

**Figure 2:**
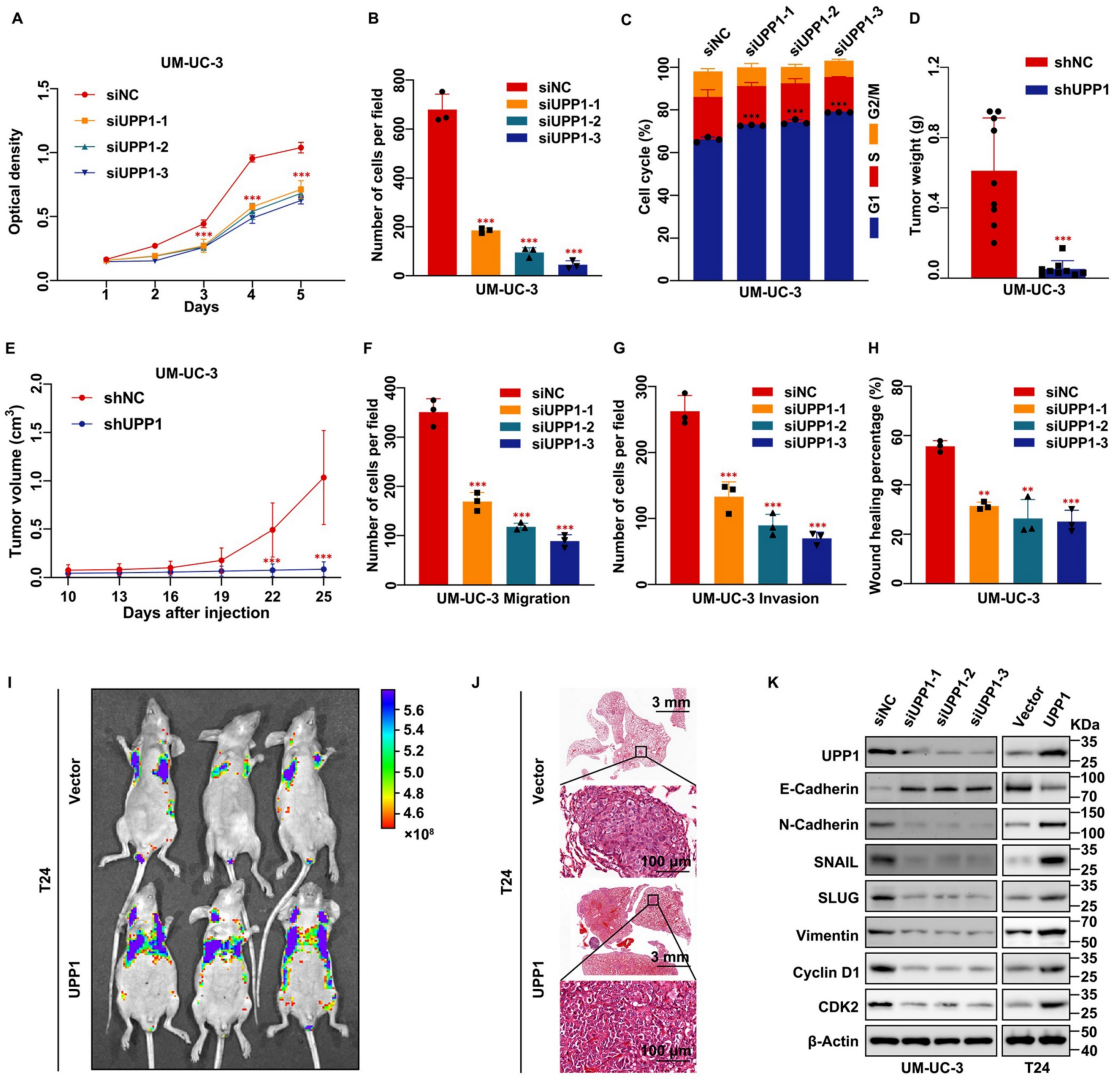
***UPP1* deficiency suppresses BLCA cell proliferation, metastasis, and invasion* in vitro* and* in vivo*. (A)** MTT assay results revealed that *UPP1* knockdown inhibited the proliferation of UM-UC-3 cells. **(B)** Statistical chart of the colony formation assay results with *UPP1* knockdown UM-UC-3 cells. **(C)** The statistical chart of the flow cytometric analysis revealed that *UPP1* knockdown promoted cell cycle arrest at the G1 phase. The weight **(D)** and volume **(E)** of tumors derived from shNC-transfected or shUPP1-transfected UM-UC-3 cells. **(F-G)** Statistical chart of the results of the transwell assay with *UPP1* knockdown UM-UC-3 cells. **(H)** Statistical chart of the wound healing assay results with *UPP1* knockdown UM-UC-3 cells. Fluorescence intensity **(I)** and H&E staining **(J)** of the lung from the BALB/c nude mouse metastasis model injected with Vector-transfected and UPP1-transfected T24 cells. **(K)** Immunoblotting analysis revealed that *UPP1* knockdown downregulated the expression of EMT-related and cell cycle-related proteins in UM-UC-3 cells. UPP1 overexpression promoted the expression of these proteins in T24 cells. *: *p* < 0.05; **: *p* < 0.01; ***: *p* < 0.001; ns: not significant (*p* > 0.05).

MTT assay results showed that the proliferation rate of *UPP1* knockdown BLCA cells was significantly lower than that of control cells (Fig. 2A and Supplementary Fig. S2E). Additionally, the viability of UPP1-overexpressing BLCA cells was higher than that of control cells (Supplementary Fig. S2F-G). The colony formation assay results revealed that the size and number of colonies formed by *UPP1* knockdown BLCA cells were lower than those formed by control cells (Fig. 2B and Supplementary Fig. S2H-I). Meanwhile, the size and number of colonies formed by UPP1-overexpressing BLCA cells were higher than those of colonies formed by control cells (Supplementary Fig. S2J-K). Flow cytometric analysis demonstrated that the cell cycle of *UPP1* knockdown cells was arrested at the G1 phase (Fig. 2C, Supplementary Figs. S2L and S3A-B). Meanwhile, UPP1 overexpression promoted cell cycle transition (Supplementary Figs. S2M and S3C-D).

To further verify the function of UPP1 in cell proliferation *in vivo*, a stable *UPP1-*knockdown UM-UC-3 cell line and a stable UPP1-overexpressing T24 cell line were established using lentiviral transduction. *UPP1* knockdown and overexpression efficiency was determined using qRT-PCR and immunoblotting (Supplementary Fig. S4A-D). shNC-transfected or shUPP1-transfected UM-UC-3 cells and Vector-transfected or UPP1-transfected T24 cells were injected into the subcutaneous tissue of BALB/c nude mice. The tumor size was regularly measured using a vernier caliper to determine tumor growth. The tumor was dissected at the specified time. The growth rate and weight of tumors derived from shUPP1-transfected UM-UC-3 cells were significantly lower than those derived from shNC-transfected UM-UC-3 cells (Fig. 2D-E and Supplementary Fig. S4E). Additionally, the tumors derived from UPP1-transfected T24 cells grew faster than those derived from Vector-transfected T24 cells (Supplementary Fig. S4F-H).

Transwell assay results showed that *UPP1* knockdown significantly inhibited BLCA cell migration and invasion (Fig. 2F-G and Supplementary Fig. S5A-C). In contrast, UPP1 overexpression promoted the migration and invasion of BLCA cells (Supplementary Fig. S5D-F). The wound healing assay results were consistent with those of the transwell assay (Fig. 2H and Supplementary Fig. S5G-J). Next, an experimental tumor lung metastasis model was established by injecting nude mice aged six weeks with shNC-transfected or shUPP1-transfected UM-UC-3 cells and Vector-transfected or UPP1-transfected T24 cells via the tail vein. The GFP fluorescence intensity in mice injected with shUPP1-transfected UM-UC-3 cells was lower than that in mice injected with shNC-transfected UM-UC-3 cells (Supplementary Fig. S4I), and the GFP fluorescence intensity in mice injected with UPP1-transfected T24 cells was higher than that in mice injected with Vector-transfected T24 cells (Fig. 2I). H&E staining also supported these results (Fig. 2J and Supplementary Fig. S4J). Finally, immunoblotting revealed that UPP1 modulation altered the expression levels of biomarkers related to the cell cycle and epithelial-mesenchymal transition (EMT) (Fig. 2K and Supplementary Fig. S5K).

### *UPP1* knockdown suppresses tumorigenesis and promotes BLCA cell apoptosis and ROS generation by downregulating the AKT signaling pathway

**Figure 3:**
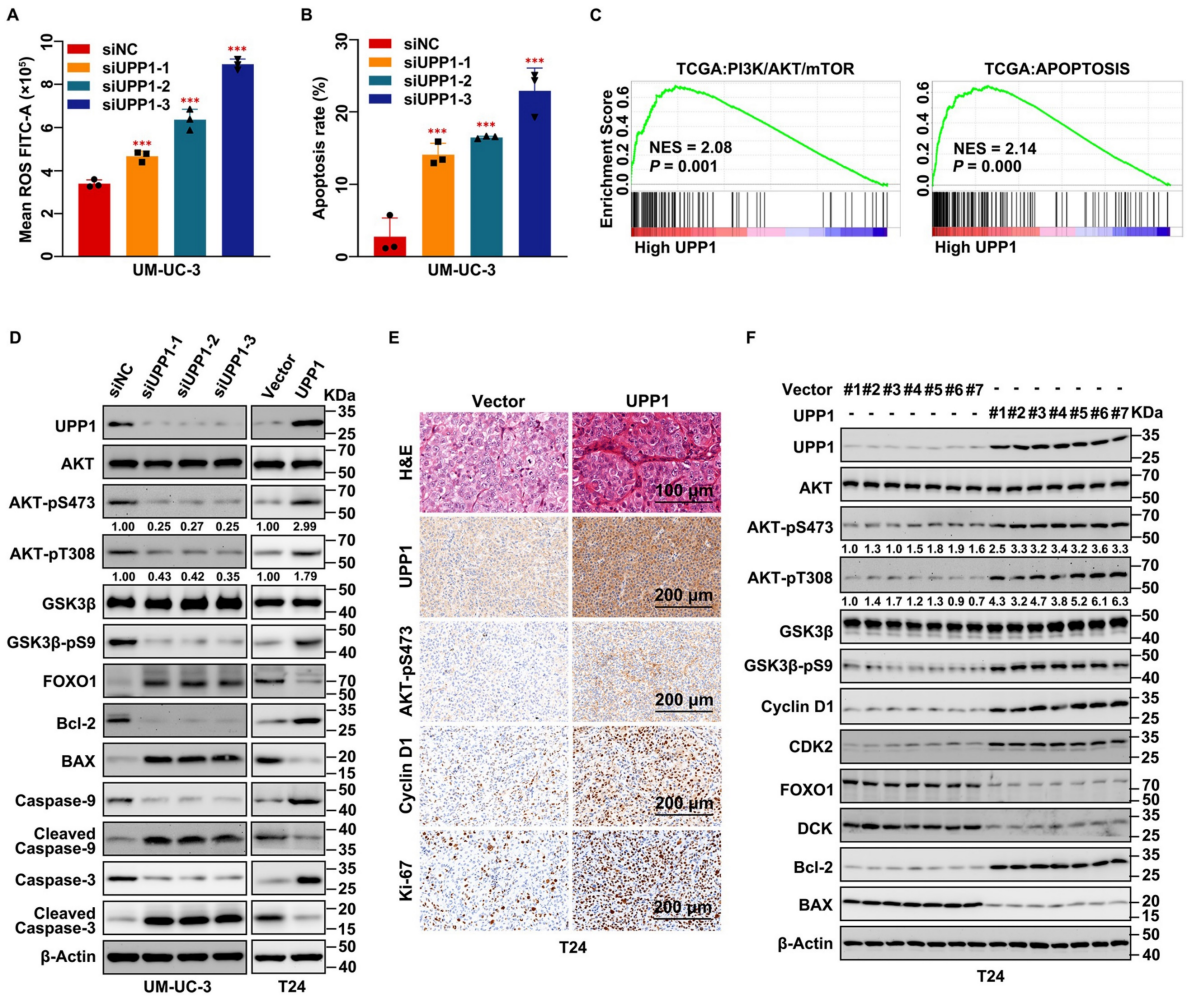
***UPP1* knockdown upregulates cellular ROS levels and promotes apoptosis in BLCA through the AKT signaling pathway *in vitro* and* in vivo*. (A)** Flow cytometric analysis revealed that *UPP1* knockdown upregulated ROS levels in UM-UC-3 cells. **(B)** Statistical chart of the apoptosis rates of UM-UC-3 cells evaluated using flow cytometry. **(C)** GSEA based on TCGA dataset. **(D)** Immunoblotting analysis revealed that *UPP1* knockdown suppressed the phosphorylation of AKT at Ser473 and Thr308, downregulated anti-apoptotic proteins, and upregulated pro-apoptotic proteins in UM-UC-3 cells. UPP1 overexpression reversed the expression trend of these proteins in T24 cells.** (E)** H&E and IHC staining of subcutaneous Vector-transfected and UPP1-transfected T24 cell-derived xenograft tumors. **(F)** Immunoblotting indicates that UPP1 overexpression could promote AKT phosphorylation and inhibit apoptosis of BLCA cells *in vivo*. *: *p* < 0.05; **: *p* < 0.01; ***: *p* < 0.001; ns: not significant (*p* > 0.05).

Next, the regulatory effects of UPP1 on apoptosis in BLCA were examined. Flow cytometric analysis was used to determine the cellular ROS level. The cellular ROS levels were upregulated in *UPP1* knockdown cells (Fig. 3A and Supplementary Fig. S6A-C) and downregulated in UPP1-overexpressing cells (Supplementary Fig. S6D-F) compared with those in control cells. Flow cytometric analysis revealed that the percentage of apoptotic cells in the *UPP1* knockdown group was higher than that in the control group (Fig. 3B and Supplementary Fig. S6G-I).

GSEA revealed that AKT, apoptosis, and EMT-related pathways were upregulated (Fig. 3C and Supplementary Fig. S7A). GSVA revealed that the expression of UPP1 was significantly related to PI3K/AKT/mTOR, apoptosis, ROS, and EMT-related signaling pathways (Supplementary Fig. S7B). Immunoblotting analysis demonstrated that *UPP1* knockdown inhibited the phosphorylation of AKT and GSK3β, resulting in the downregulation of the pro-survival factor Bcl-2 33 and the upregulation of FOXO1 and the pro-apoptotic factor BAX 34 (Fig. 3D and Supplementary Fig. S7C). *UPP1* knockdown downregulated Caspase 9 and Caspase 3 levels but upregulated the Cleaved Caspase 9 and Cleaved Caspase 3 levels. The expression patterns of these proteins in UPP1-overexpressing cells contrasted with those in *UPP1* knockdown cells (Fig. 3D and Supplementary Fig. S7C).

Next, H&E and IHC staining of dissected tumors indicated that the number of cells in the tumors derived from shUPP1-transfected UM-UC-3 cells was lower than that in the tumors derived from shNC-transfected UM-UC-3 cells (Supplementary Fig. S7D). Additionally, the proliferation-related biomarkers Cyclin D1, Ki-67, and AKT-pS473 were downregulated in the tumors derived from shUPP1-transfected UM-UC-3 cells (Supplementary Fig. S7D). In contrast, the number of cells in the tumor derived from UPP1-transfected T24 cells was higher than that in the tumor derived from Vector-transfected T24 cells (Fig. 3E), and the pro-proliferative biomarkers were upregulated in the tumor derived from UPP1-transfected T24 cells (Fig. 3E). Total protein was extracted from the subcutaneous tumors derived from UM-UC-3 cells (shNC-transfected and shUPP1-transfected) and T24 cells (Vector-transfected and UPP1-transfected). Immunoblotting revealed that the AKT signaling pathway was downregulated, and the expression of pro-apoptosis-related biomarkers was upregulated in the tumors derived from shUPP1-transfected cells (Supplementary Fig. S7E), which was consistent with the results of the *in vitro* experiments. In contrast, the AKT signaling pathway was upregulated and pro-apoptosis-related biomarkers were downregulated in UPP1-transfected T24 cells (Fig. 3F). These results suggest that *UPP1* knockdown promotes apoptosis by downregulating the AKT signaling pathway.

**Figure 4:**
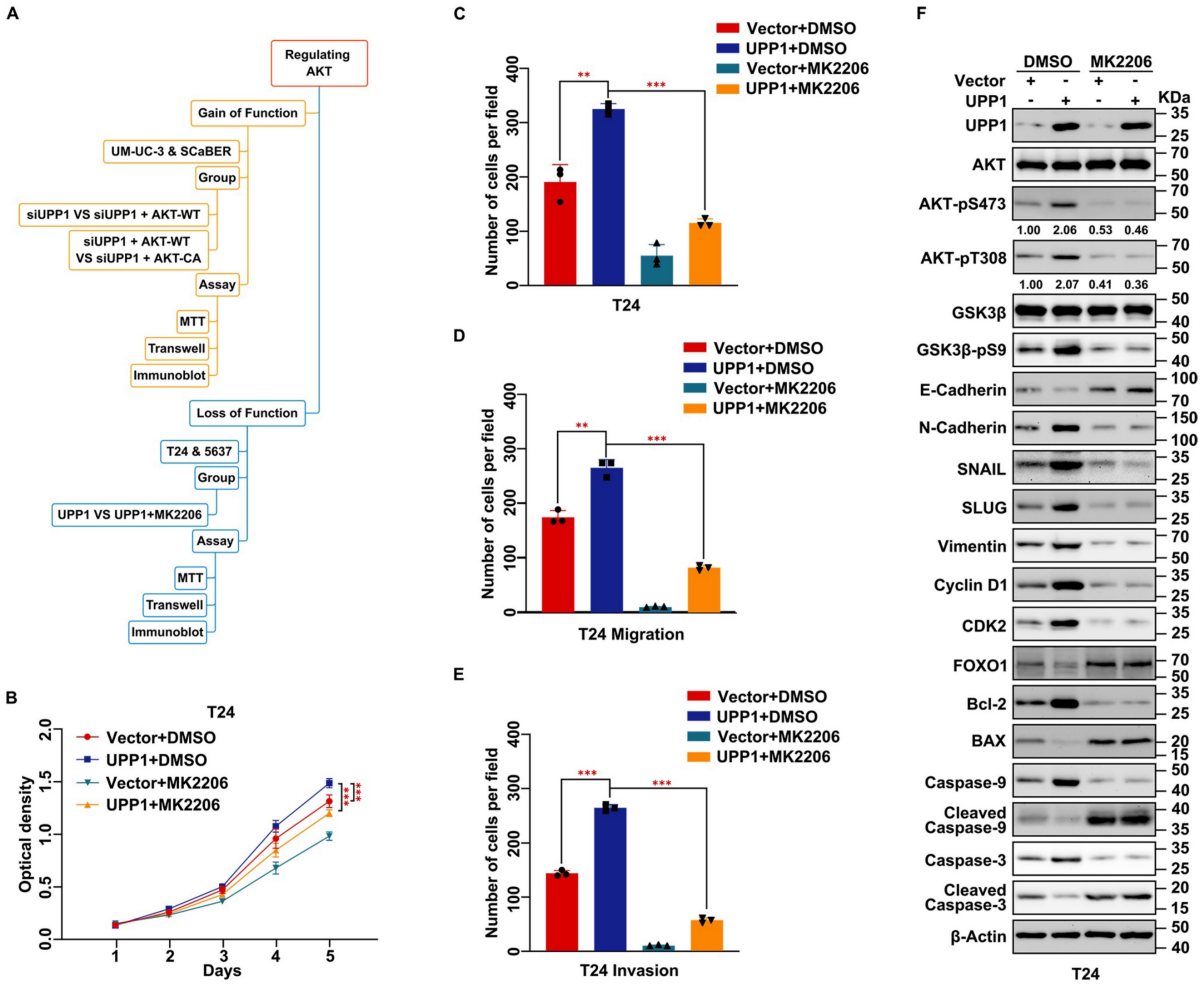
** Inhibition of AKT rescues the stimulating effect of UPP1 overexpression in BLCA cells. (A)** Frame diagram of rescue experiments. **(B)** MTT assay results revealed that treatment with MK2206 (10 μM) suppressed the UPP1-induced upregulation of T24 cell proliferation. **(C)** The colony formation assay results revealed that MK2206 (10 μM) mitigated the UPP1-induced upregulation of T24 cell proliferation. **(D-E)** The statistical chart of the transwell assay results indicates that MK2206 (10 μM) suppressed the UPP1-induced upregulation of migration and invasion in T24 cells.** (F)** Immunoblotting analysis revealed that UPP1-induced AKT activation was mitigated upon treatment with MK2206 (10 μM). *: *p* < 0.05; **: *p* < 0.01; ***: *p* < 0.001; ns: not significant (*p* > 0.05).

To further verify whether UPP1 exerts regulatory effects on the biological function of BLCA via the AKT signaling pathway, cells were treated with the AKT-specific inhibitor MK2206 or transfected with the AKT plasmid. A constitutively active plasmid AKT-CA was constructed by mutating Thr308 and Ser473 to Asp to simulate phosphorylated AKT 35, 36 (Fig. 4A). MTT assay results showed that MK2206 significantly decreased the viability of UPP1-overexpressing BLCA cells (Fig. 4B and Supplementary Fig. S8A). The results of the colony formation assay were consistent with those of the MTT assay (Fig. 4C and Supplementary Fig. S8B-C). Meanwhile, transwell assay results indicated that MK2206 treatment significantly inhibited the migration and invasion of UPP1-overexpressing BLCA cells (Fig. 4D-E and Supplementary Fig. S8D-E). Immunoblotting analysis demonstrated that the phosphorylation of AKT (Ser473 and Thr308) and the expression of downstream EMT-related biomarkers were downregulated. In contrast, the expression levels of pro-apoptotic biomarkers were upregulated (Fig. 4F and Supplementary Fig. S8F).

**Figure 5:**
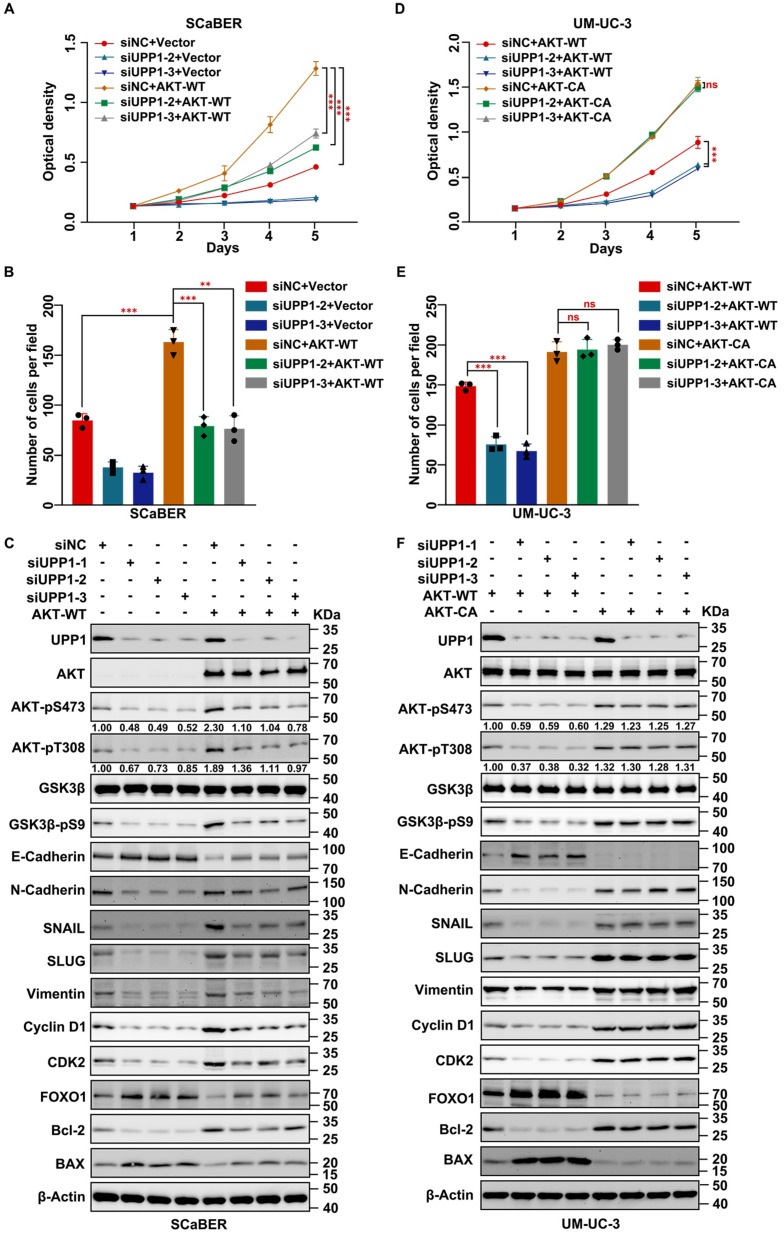
** AKT overexpression rescues the inhibitory effect of *UPP1* knockdown in BLCA cells. (A)** MTT assay results with *UPP1* knockdown and AKT-WT-overexpressing SCaBER cells. **(B)** The results of the transwell assay with *UPP1* knockdown and AKT-WT-overexpressing SCaBER cells. **(C)** Immunoblotting analysis of the effect of *UPP1* knockdown and AKT-WT overexpression on the expression of related proteins in SCaBER cells. **(D)** The results of the MTT assay examine the effect of AKT-WT and AKT-CA overexpression in *UPP1* knockdown UM-UC-3 cells. **(E)** The results of the transwell assay examining the effect of AKT-WT and AKT-CA overexpression in *UPP1* knockdown UM-UC-3 cells. **(F)** Immunoblotting analysis of the expression of related proteins in *UPP1* knockdown, AKT-WT-overexpressing, and AKT-CA-overexpressing UM-UC-3 cells. *: *p* < 0.05; **: *p* < 0.01; ***: *p* < 0.001; ns: not significant (*p* > 0.05).

Next, AKT-WT was overexpressed in *UPP1* knockdown cells. MTT assay results suggested that AKT-WT overexpression partially restored the impaired proliferation of *UPP1* knockdown BLCA cells (Fig. 5A and Supplementary Fig. S9A). Consistently, the transwell assay results showed that AKT-WT overexpression partially restored the impaired migration of *UPP1* knockdown BLCA cells (Fig. 5B and Supplementary Fig. S9B-C). Immunoblotting revealed that AKT-WT overexpression partially mitigated the *UPP1* knockdown-induced downregulation of AKT phosphorylation and downstream cell cycle- and EMT-related biomarkers. In contrast, AKT-WT overexpression partially mitigated the *UPP1* knockdown-mediated upregulation of pro-apoptosis-related biomarkers (Fig. 5C and Supplementary Fig. S10A). Additionally, BLCA proliferation (Fig. 5D and Supplementary Fig. S9D) and migration (Fig. 5E and Supplementary Fig. S9E-F) in AKT-CA-overexpressing *UPP1* knockdown cells were fully rescued when compared with those of AKT-WT-overexpressing cells. Immunoblotting also confirmed these conclusions (Fig. 5F and Supplementary Fig. S10B). These findings indicate that UPP1 regulates the biological function of BLCA by activating the AKT signaling pathway.

### UPP1 promotes AKT dual-site phosphorylation by interacting with the C-terminus of AKT

**Figure 6:**
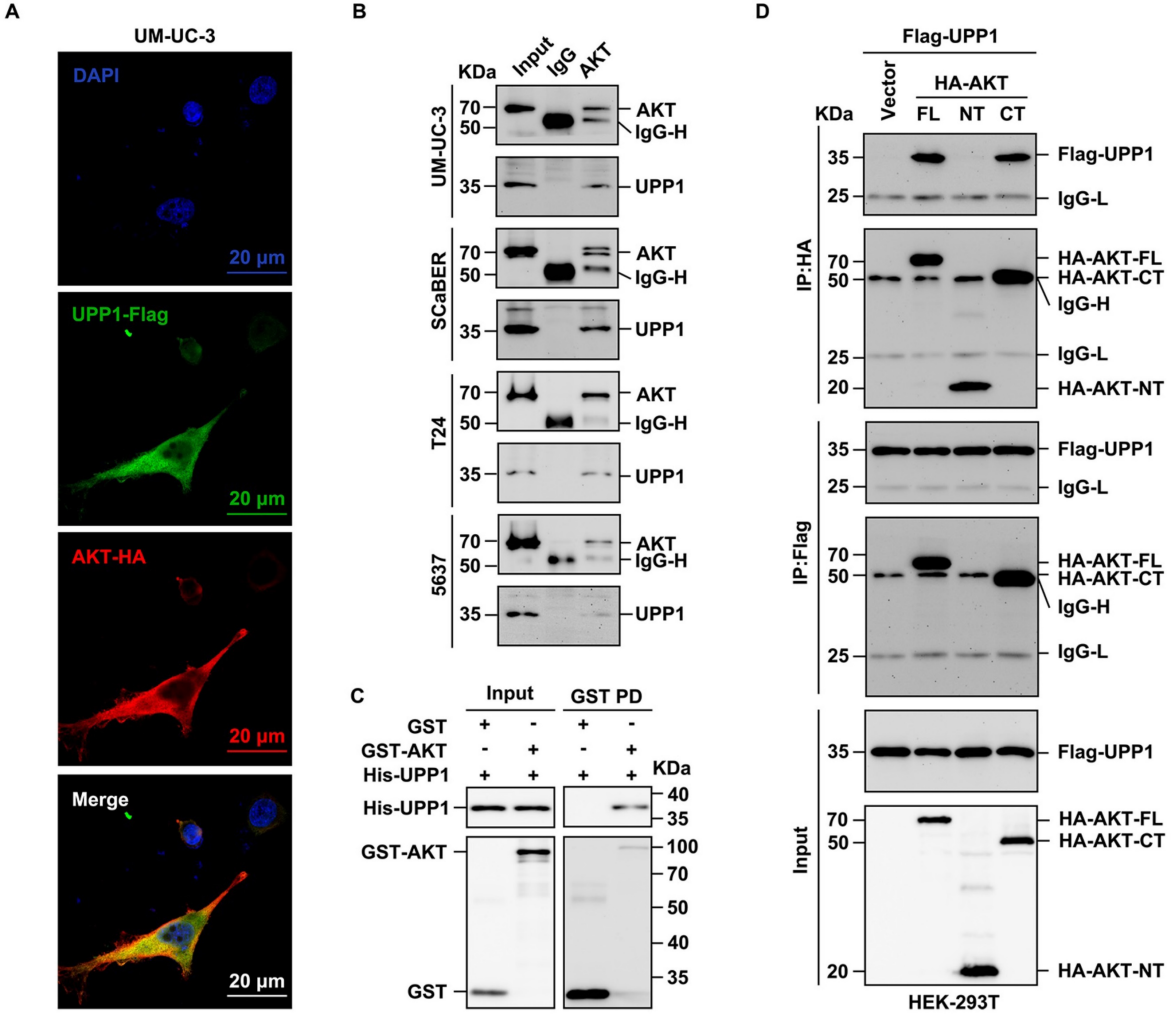
** UPP1 interacts with the C-terminus of AKT. (A)** Immunofluorescence analysis of the cytoplasmic co-localization of UPP1-Flag and AKT-HA in UM-UC-3 cells. **(B)** Endogenous co-IP analysis indicates that UPP1 interacted with AKT in UM-UC-3, SCaBER, T24, and 5637 cell lines. **(C)** GST pull-down assay results revealed that UPP1 interacted with AKT* in vitro*. **(D)** Exogenous co-IP analysis demonstrated that UPP1 interacted with the C-terminus of AKT in HEK-293T cells.

Next, the biochemical mechanism and specific interaction domains were identified using the co-IP assay (Supplementary Fig. S11A). The results of the exogenous co-IP assay demonstrated a definite interaction between UPP1 and AKT in HEK-293T cells (Supplementary Fig. S11B). Immunofluorescence analysis revealed that UPP1 co-localized with AKT in UM-UC-3 cell cytoplasm (Fig. 6A). Meanwhile, the results of the endogenous co-IP assay demonstrated that UPP1 interacted with AKT in multiple BLCA cell lines (Fig. 6B). GST pull-down analysis showed direct binding between recombinant UPP1 and recombinant AKT *in vitro* (Fig. 6C). Truncated AKT-NT and AKT-CT plasmids were constructed, and analysis of the interaction between UPP1 and truncated AKT demonstrated that UPP1 could interact with AKT-CT (Fig. 6D).

**Figure 7:**
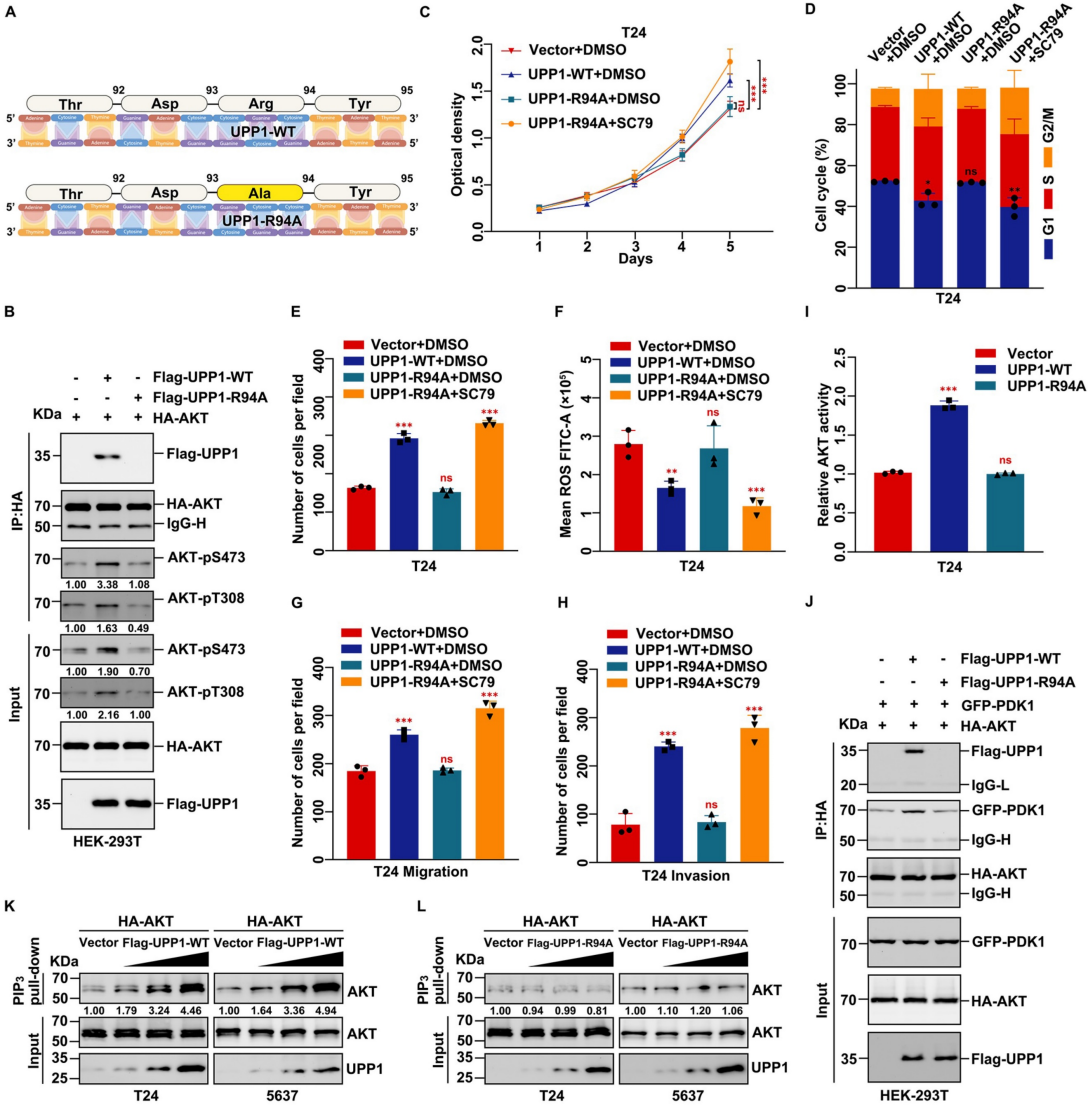
** UPP1 promotes AKT activation through direct interaction. (A)** Schematic for site mutation of *UPP1*. **(B)** Quantitative exogenous co-IP analysis indicates that UPP1-R94A overexpression did not promote AKT phosphorylation in HEK-293T cells. **(C)** The results of MTT assay with T24 cells overexpressing UPP1-WT or UPP1-R94A. SC79 (20 μM) promoted the proliferation of T24 cells. **(D)** UPP1-WT or UPP1-R94A overexpression did not promote cell cycle transition in T24 cells. SC79 (20 μM) promoted cell cycle transition in T24 cells. **(E)** The results of the colony formation assay with T24 cells overexpressing UPP1-WT or UPP1-R94A. SC79 (20 μM) increased the colony numbers of T24 cells. **(F)** ROS levels in cells overexpressing UPP1-WT or UPP1-R94A. SC79 (20 μM) significantly downregulated the intercellular ROS level. **(G-H)** The results of the transwell assay with T24 cells overexpressing UPP1-WT or UPP1-R94A. SC79 (20 μM) promoted the metastasis of T24 cells. **(I)** The relative AKT activity in T24 cells overexpressing UPP1-WT or UPP1-R94A.** (J)** UPP1-WT promoted the binding of AKT to PDK1 in HEK-293T cells.** (K)** UPP1-WT facilitated phosphatidylinositol 3,4,5-triphosphate (PIP_3_) recruitment to AKT. **(L)** UPP1-R94A did not promote PIP_3_ recruitment to AKT. *: *p* < 0.05; **: *p* < 0.01; ***: *p* < 0.001; ns: not significant (*p* > 0.05).

To further investigate the mechanism underlying the interaction between UPP1 and AKT, a mutant UPP1 overexpression plasmid was constructed. Previous studies have analyzed the molecular structure of UPP1 and reported that Arg94 is a critical residue that binds to phosphate. Alteration of Arg94 modulates the biological function of UPP1 25, 37, 38. Thus, Arg94 was mutated to Ala94 to construct the mutant plasmid UPP1-R94A (Fig. 7A). The effects of UPP1-R94A overexpression were compared with those of UPP1-WT. The interaction between AKT and mutant UPP1-R94A was analyzed by co-IP assay. UPP1-R94A did not interact with AKT-FL (Supplementary Fig. S11C). Next, quantitative exogenous co-IP analysis was performed to clarify the phosphorylation level of AKT. When equal amounts of AKT were immunoprecipitated, the phosphorylation levels of AKT in immunoprecipitated and input samples were upregulated upon UPP1-WT overexpression but did not markedly change upon UPP1-R94A overexpression. The AKT phosphorylation levels in UPP1-R94A-overexpressing cells were similar to those in the control group (Fig. 7B). These results suggest that Arg94 is an AKT-binding site and essential for the phosphorylation of AKT.

The effects of UPP1-R94A on biological functions were examined. MTT assay results demonstrated that the proliferation of UPP1-R94A-overexpressing cells was similar to that of the control group (Fig. 7C and Supplementary Fig. S11D). Treatment with SC79, an AKT-specific activator, promoted cell proliferation (Fig. 7C and Supplementary Fig. S11D). UPP1-R94A overexpression did not promote cell cycle transition (Fig. 7D and Supplementary Figs. S11E and S12A-B). Treatment with SC79 promoted the UPP1-R94A-overexpressing cell cycle transition process (Fig. 7D and Supplementary Figs. S11E and S12A-B). Furthermore, the colony formation assay results indicated that UPP1-R94A overexpression did not increase the colony number or size (Fig. 7E and Supplementary Fig. S11F-G). UPP1-R94A overexpression did not downregulate the cellular ROS level. Treatment with SC79 downregulated the ROS levels of UPP1-R94A-overexpressing cells (Fig. 7F and Supplementary Fig. S11H-J). The results of the transwell assay showed that UPP1-R94A overexpression did not promote BLCA cell migration and invasion. However, SC79 treatment strengthened the invasion and migration of UPP1-R94A-overexpressing cells (Fig. 7G-H and Supplementary Fig. S11K-M).

Immunoblotting analysis revealed that UPP1-R94A did not activate the AKT signaling pathway. In contrast, SC79 activated the AKT signaling pathway in UPP1-R94A-overexpressing cells (Supplementary Fig. S13A). To rule out the interference of endogenous UPP1 with the experimental results, *UPP1* was knocked down, and UPP1-WT or UPP1-R94A was overexpressed. The immunoblotting results were consistent with the previous results (Supplementary Fig. S13B). These findings show that UPP1 promotes BLCA cell proliferation, migration, and invasion and inhibits ROS generation and apoptosis in BLCA by activating the AKT signaling pathway by interacting with the C-terminus. In contrast, UPP1-R94A did not exert these functions.

UPP1, a phosphorylase, has no structural basis to support serine/threonine kinase activity against AKT. Thus, the mechanism by which UPP1 mediates the phosphorylation of AKT and the role of the UPP1-AKT interaction were examined. In particular, the effect of artificial modulation of UPP1 expression in BLCA cells on the expression of PI3K, PTEN, PDK1, and PDK2, which are critical proteins that regulate AKT phosphorylation, was examined. Immunoblotting analysis revealed that *UPP1* downregulation or overexpression did not affect the protein levels of PI3K, phosphorylated PI3K, PTEN, PDK1, and PDK2 (Supplementary Fig. S14A). The activity of AKT was quantified using the AKT kinase activity kit. *UPP1* knockdown inhibited the activity of AKT (Supplementary Fig. S14B-C). UPP1 overexpression upregulated AKT activity, but UPP1-R94A overexpression did not promote the phosphorylation of AKT (Fig. 7I and Supplementary Fig. S14D).

Next, PDK1, PDK2, and PTEN overexpression vectors were constructed, and a series of co-IP assays were performed to explore the relationship between UPP1 and these proteins. Exogenous co-IP analysis revealed that AKT interacted with PDK1 and PDK2 in HEK-293T cells (Supplementary Fig. S14E-F). Moreover, UPP1 could not interact with PTEN (Supplementary Fig. S14G). Meanwhile, UPP1 and UPP1-R94A interacted with PDK1 and PDK2 (Supplementary Fig. S15A-B). The results of the quantitative exogenous co-IP experiments demonstrated that UPP1, but not UPP1-R94A, facilitates AKT binding to PDK1 and PDK2 (Fig. 7J and Supplementary Fig. S15C). These results suggest that UPP1 promotes AKT activation by facilitating the binding of AKT to PDK1 and PDK2, which depends on the interaction between UPP1 and AKT.

As the activation of AKT is associated with its binding to PIP_3_, a PIP_3_ pull-down assay was performed to elucidate the potential mechanisms. UPP1 overexpression, but not UPP1-R94A overexpression, increased the interaction between AKT and PIP_3_ (Fig. 7K-L). These results suggest that UPP1 promotes the phosphorylation of AKT by facilitating PIP_3_ recruitment to AKT, which depends on the interaction between UPP1 and AKT.

Furthermore, SC79 was demonstrated to activate AKT and rescue the malignant phenotype of UPP1-R94A-overexpressing BLCA cells. SC79 activates AKT in the cytoplasm and inhibits AKT membrane translocation 39-41. Therefore, an exogenous co-IP assay was implemented to investigate the effect of SC79 on the binding between UPP1-R94A and AKT. UPP1-R94A did not interact with AKT in the presence of SC79 (Supplementary Fig. S15D). These results suggest that UPP1-R94A is not involved in the SC79-mediated activation of AKT.

Thus, this study demonstrates that UPP1 interacts with the C-terminus of AKT to promote the BLCA malignant phenotype. UPP1 activates the AKT signaling pathway by promoting the binding of AKT to PDK1 and PDK2 and facilitating the recruitment of PIP_3_ to AKT, which depends on the binding of UPP1 to AKT.

### UPP1 promotes gemcitabine resistance in BLCA through the AKT/FOXO1/DCK signaling pathway

**Figure 8:**
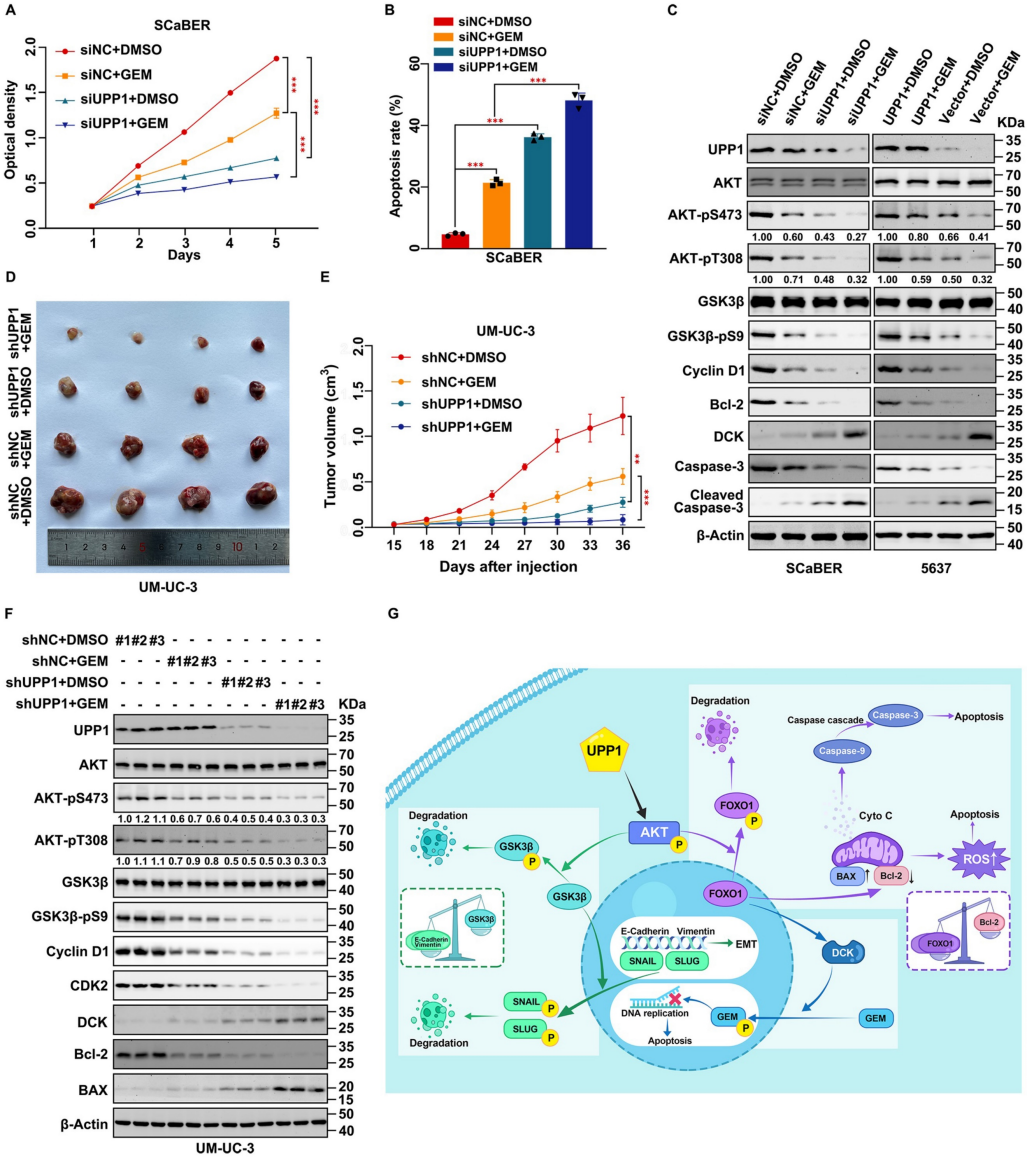
***UPP1* knockdown promotes gemcitabine chemosensitivity through the AKT/FOXO1/DCK signaling pathway. (A)** MTT assay results revealed that *UPP1* knockdown promoted gemcitabine chemosensitivity in SCaBER cells. **(B)** *UPP1* knockdown significantly increased the apoptosis rate in SCaBER cells. **(C)** Immunoblotting analysis revealed that gemcitabine significantly downregulated AKT phosphorylation and upregulated pro-apoptotic protein expression in *UPP1* knockdown SCaBER cells. UPP1 overexpression suppressed the gemcitabine-induced inhibition of AKT phosphorylation and downregulated pro-apoptotic protein expression in gemcitabine-treated 5637 cells. The effect of gemcitabine on the volume **(D)** and viability **(E)** of shUPP1-transfected and shNC-transfected UM-UC-3 cell-derived xenograft tumors. **(F)** Analysis of the protein levels in xenograft tumors of different groups. **(G)** Schematic showing the mechanism by which UPP1 regulates biological status via the AKT signaling pathway. *: *p* < 0.05; **: *p* < 0.01; ***: *p* < 0.001; ns: not significant (*p* > 0.05).

Gemcitabine is a pyrimidine analog. Intravesical gemcitabine is a standard, effective, and safe therapy for NMIBC and MIBC 42. The beneficial effects of gemcitabine in patients during postoperative recurrence and progression-free survival were higher than those of mitomycin 43. Downregulation of the AKT signaling pathway can increase gemcitabine sensitivity in BLCA 44, 45. Therefore, based on our previous findings, experiments were performed to explore the clinical value of increasing gemcitabine chemosensitivity. *UPP1* knockdown in multiple BLCA cells significantly decreased the half-maximal inhibitory concentration of gemcitabine, and opposite results were found in UPP1 overexpression cells (Supplementary Fig. S16A-D). MTT assay results indicated that *UPP1* knockdown increased gemcitabine chemosensitivity in SCaBER (300 nM) and UM-UC-3 (400 nM) cells (Fig. 8A and Supplementary Fig. S16E). In contrast, UPP1 overexpression promoted drug tolerance in T24 (200 nM) and 5637 (400 nM) cells (Supplementary Fig. S16F-G). Gemcitabine significantly increased the apoptotic rate in *UPP1* knockdown cells (Fig. 8B, and Supplementary Figs. S16H and S17A-B). Immunoblotting analysis revealed that Cleaved Caspase 3, an apoptosis marker, was upregulated in *UPP1* knockdown cells. *UPP1* knockdown upregulated the expression of deoxycytidine kinase (DCK), a critical enzyme that can phosphorylate gemcitabine to exert pharmacological effects in the nucleus. Consistent with previous results, UPP1 overexpression suppressed the expression levels of Cleaved Caspase 3 and DCK (Fig. 8C and Supplementary Fig. S16I).

To further explore the function of UPP1 in gemcitabine tolerance *in vivo*, a tumor formation assay was performed by injecting UM-UC-3 cells (shNC-transfected and shUPP1-transfected) and T24 cells (Vector-transfected and UPP1-transfected) into nude mouse subcutaneous tissue. After allowing the tumors to grow for 15 days, the mice were intraperitoneally administered gemcitabine (50 mg/kg bodyweight) or DMSO twice a week. The tumor weight in the gemcitabine-treated *UPP1* knockdown group was significantly lower than that in the group treated with gemcitabine (Fig. 8D and Supplementary Fig. S18A). Cell viability in the gemcitabine-treated *UPP1* knockdown group was significantly weaker than that in the group treated with gemcitabine (Fig. 8E). H&E staining revealed that the cell density in the tumors of the gemcitabine-treated *UPP1* knockdown group was lower than that in the tumors of the other three groups (Supplementary Fig. S18B). IHC analysis demonstrated that AKT-pS473, Cyclin D1, and Ki-67 were downregulated (Supplementary Fig. S18B). Immunoblotting of xenograft tumors indicated that gemcitabine inhibited the phosphorylation of AKT (Fig. 8F). In contrast, the tumor weight and cell viability in the gemcitabine-treated UPP1-overexpressing group were significantly higher than those in the group treated with gemcitabine (Supplementary Fig. S18C-E). H&E staining revealed that the cell density in the tumors of the gemcitabine-treated UPP1-overexpressing group was higher than that in the tumors of the other three groups (Supplementary Fig. S18G). IHC analysis demonstrated that AKT-pS473, Cyclin D1, and Ki-67 were downregulated (Supplementary Fig. S18G). Immunoblotting of xenograft tumors indicated that gemcitabine inhibited the phosphorylation of AKT (Supplementary Fig. S18F). The combination of gemcitabine treatment and UPP1 overexpression markedly activated the AKT signaling pathway and promoted BLCA cell gemcitabine resistance.

Next, the specific downstream mechanisms by which UPP1 regulates DCK expression were examined. A previous study by our team confirmed a positive correlation between DCK and FOXO1 expression 46. *UPP1* knockdown promoted the expression of DCK, whereas UPP1 overexpression downregulated the expression of DCK (Supplementary Fig. S19A). Treatment with the AKT inhibitor MK2206 mitigated the inhibitory effects of UPP1 overexpression on DCK (Supplementary Fig. S19B). AKT-WT overexpression partially inhibited the *UPP1* knockdown-induced upregulation of DCK (Supplementary Fig. S19C). Meanwhile, AKT-CA overexpression completely inhibited the protein level of DCK (Supplementary Fig. S19D). Additionally, UPP1-R94A overexpression did not suppress the expression of DCK (Supplementary Fig. S19E). Furthermore, UPP1-R94A overexpression suppressed the expression of DCK in *UPP1* knockdown cells (Supplementary Fig. S19F). These findings indicate that UPP1 promotes gemcitabine resistance in BLCA through the AKT/FOXO1/DCK signaling pathway.

In summary, our study indicates that UPP1 activates the AKT signaling pathway by facilitating AKT binding to PDK1 and PDK2 and promoting the interaction between PIP_3_ and AKT in BLCA cells, which depends on the binding of UPP1 to the AKT C-terminus. UPP1 promotes BLCA cell proliferation via the AKT/GSK3β/Cyclin D1 signaling pathway, facilitates BLCA cell metastasis through the AKT/GSK3β/SNAIL signaling pathway, inhibits BLCA cell apoptosis and ROS generation by the AKT/FOXO1/Bcl-2 signaling pathway, and improves BLCA cell gemcitabine resistance via the AKT/FOXO1/DCK signaling pathway (Fig. 8G).

## Discussion

UPP1, which is involved in pyrimidine metabolism, regulates uridine homeostasis and promotes pyrimidine salvage 47. Previous studies have reported that UPP1 is involved in the pathogenesis of colon cancer 48, glioma 26, breast cancer 49, thyroid cancer 50, oral squamous cell carcinoma 51, pancreatic cancer 52, and lung adenocarcinoma 53. The oncogenic functions of UPP1 are suggested to mediate tumorigenesis. UPP1 overexpression is associated with poor prognosis and cancer progression 54. Cao *et al*. detected tumors in multiple organs of a *Upp1*^-/-^ murine model and reported that *Upp1* deficiency promotes DNA damage and sequentially activates the ATM/CHK2/p53 signaling pathway 55. Wang *et al*. reported that *UPP1* deficiency inhibited glycolysis in lung adenocarcinoma progression by suppressing the expression of ENO1 and LDHA 53. A recent study demonstrated that pancreatic ductal adenocarcinoma utilizes uridine as a crucial compensatory fuel to meet metabolic needs under nutrient-deficient conditions and that this is mainly mediated by UPP1 56. In addition, small molecule inhibitors of UPP1 have been reported in clinical trials. 5-FU, a widely used chemotherapy drug for treating various types of cancer globally 57, has limited clinical application due to dose-dependent cytotoxicity 58. It has been reported that *UPP1* deficiency leads to the accumulation of uridine, which can reduce 5-FU toxicity in normal tissues 59, and BAU is a UPP1-specific inhibitor that protects normal tissues from 5-FU toxicity 60-62. Zhao et al. found that the glutaminase 1 (GLS1) inhibitor CB-839 could effectively inhibit colorectal cancer with PIK3CA mutations 63. In addition, CB-839 upregulated the expression of UPP1 in various transplantation tumor models, strengthening the inhibitory effect of 5-FU on PIK3CA-mutant colorectal cancer 63. da Silva EFG et al. demonstrated for the first time the ability of CPBMF65 to inhibit the proliferation of HepG2 cells by blocking the cell cycle and promoting cellular senescence 64. Based on its *in vitro* antitumor activity and low toxicity in normal cells, CPBMF65 may be a candidate for future *in vivo* therapeutic studies in hepatocellular carcinoma.

However, previous studies examining the role of UPP1 in tumorigenesis have mainly focused on metabolism and DNA damage. Limited studies have reported the role of UPP1 in BLCA. As the p53-encoding gene is extensively mutated in BLCA 65, we hypothesized the presence of a distinct mechanism through which UPP1 promotes tumorigenesis in BLCA. This study reported that BLCA expression was upregulated and positively correlated with tumor malignancy. Kaplan-Meier survival curves revealed that UPP1 represented a poor prognosis in patients with BLCA and might be a potential biomarker.

In this study, UPP1 promoted BLCA cell proliferation, metastasis, and cell cycle transition and exerted protective effects against ROS stress-induced apoptosis through the AKT signaling pathway. The primary mechanism through which AKT regulates biological functions involves the phosphorylation of various target kinases, enzymes, and transcription factors. Patients with BLCA exhibiting hyperactivated AKT are associated with enhanced proliferative and metastatic characteristics, increasing their risk of death 66. The PH domain of AKT binds to PIP_3_, promoting the membrane translocation of AKT 67. Furthermore, AKT can be activated through a classical dual phosphorylation mechanism. AKT is phosphorylated at Thr308 by PDK1 after translocation to the cell membrane 68. PDK2 then phosphorylates AKT at Ser473, resulting in complete AKT activation 69 in association with the mTOR-Rictor complex mTORC2 70. Moreover, PTEN inhibits AKT activity by dephosphorylating PIP_3_ 71. However, it is unclear if this accounts for the full activation or overactivation of AKT and if the dysregulation of downstream functions is associated with excessive AKT activation. Additionally, AKT can be phosphorylated at Ser477 and Thr479 by the cyclin-dependent kinase 2 (CDK2)/cyclin A2 complex through direct interactions with the C-terminus of AKT, which could facilitate or partially compensate for Thr308 and Ser473 phosphorylation 72.

A series of co-IP experiments investigated the relationship between UPP1 and AKT. UPP1 facilitated the malignant phenotype of BLCA by interacting with the C-terminus of AKT, whereas mutant UPP1-R94A could not interact with AKT. Furthermore, UPP1 upregulated the phosphorylation of AKT by promoting the binding of AKT to PDK1 and PDK2 and facilitating the recruitment of PIP_3_ to AKT, which was dependent on the binding of UPP1 to AKT.

GSK3β is a substrate of AKT. AKT can phosphorylate GSK3β at Ser9, inhibiting its activity. GSK3β phosphorylates various substrates and mediates multiple biological functions. Most substrates are inhibited and degraded upon phosphorylation 73. SNAIL (SNAIL-1) and SLUG (SNAIL-2), which are essential substrates of GSK3β 74, 75, belong to the SNAIL family and are critical transcription factors for EMT 76. Additionally, SNAIL and SLUG facilitate transcriptional regulation of proteins related to EMT, such as E-Cadherin, N-Cadherin, and Vimentin, promoting the transformation of epithelial cells into mesenchymal cells 77. In this study, UPP1 upregulated the SLUG, SNAIL, Vimentin, and N-Cadherin levels and suppressed the E-Cadherin levels. Additionally, some studies have reported the co-localization and interaction between UPP1 and Vimentin in NIH/3T3 and C26 cell lines 78, although the function of this interaction has not been reported. This finding may be consistent with that of this study, which reported that UPP1 promoted EMT through the AKT pathway. Thus, UPP1 promotes BLCA metastasis through the AKT/GSK3β/SNAIL signaling pathway. Furthermore, GSK3β phosphorylates Cyclin D1, a critical cell cycle-related protein, resulting in the rapid ubiquitination and degradation of Cyclin D1 79. Our study indicated that UPP1 facilitated the expression of Cyclin D1, indicating that UPP1 activates the AKT/GSK3β/Cyclin D1 signaling pathway and promotes cell cycle transition.

FOXO1, a member of the forkhead family of transcription factors, is an essential catalytic substrate of AKT. AKT-mediated phosphorylation inhibits the function of FOXO1, resulting in the translocation of FOXO1 from the nucleus to the cytoplasm 80. FOXO1 regulates cellular metabolism, the oxidative stress response, and biological activities, such as cell cycle arrest, autophagy, and apoptosis 81, 82. The anti-apoptotic protein Bcl-2, a critical substrate of FOXO1, regulates apoptosis by modulating mitochondrial permeability. Bcl-2 is localized to the outer mitochondrial membrane and inhibits the release of cytochrome C 83. The pro-apoptotic protein BAX, an essential member of the Bcl-2 family, is localized to the cytoplasm, undergoes translocation into the mitochondria upon death signal transduction, and promotes cytochrome C release 84. Cell survival requires active apoptosis inhibition, achieved by inhibiting pro-apoptotic factor expression and promoting anti-apoptotic factor expression. The transduction of cell survival signals is dependent on AKT. Activated AKT can maintain mitochondrial integrity by inhibiting FOXO1 and subsequently promoting Bcl-2 expression and inhibiting BAX expression 85, suppressing the release of cytochrome C and the activation of the downstream caspase cascade 86. *UPP1* knockdown downregulated AKT phosphorylation, Bcl-2, Caspase 9, and Caspase 3 and upregulated BAX, FOXO1, Cleaved Caspase 9, and Cleaved Caspase 3. The effects of AKT overexpression were in contrast to those of *UPP1* knockdown.

UPP1 can increase cell viability and inhibit apoptosis. These results indicate that UPP1 can regulate mitochondrial apoptosis through the AKT/FOXO1/Bcl-2/Caspase pathway. Cancer cells are exposed to metabolic stress owing to rapid growth and nutrient depletion in the tumor microenvironment. Metabolic stress can cause ROS-induced apoptosis and lead to cell death. However, cancer cells can adapt to metabolic stress by altering their metabolic pathways. AKT is a major effector involved in the metabolic stress response 87. Several studies have reported that Bcl-2 is essential for ROS adaptation in different cell types 88, 89. *UPP1* knockdown significantly upregulated ROS levels and downregulated AKT phosphorylation and Bcl-2 expression. In contrast, UPP1 overexpression significantly downregulated ROS levels and upregulated AKT phosphorylation and Bcl-2 expression. The UPP1-R94A mutant did not exert these effects. These results suggest that UPP1 can also regulate apoptosis through the AKT/FOXO1/Bcl-2/ROS pathway with some synergy with mitochondrial apoptosis. Thus, UPP1 inhibits apoptosis in BLCA cells by phosphorylating AKT to inhibit FOXO1 and upregulating Bcl-2 levels to inhibit downstream caspase and ROS levels.

Furthermore, a high recurrence rate significantly contributes to high mortality rates in patients with BLCA. Intravesical therapy is essential for treating and preventing BLCA. Gemcitabine, a nucleotide analog, is the mainstream intravesical chemotherapy drug 90. The therapeutic effects of gemcitabine, which is activated via phosphorylation, on BLCA involve the inhibition of DNA replication. The critical enzyme mediating the phosphorylation of gemcitabine is DCK 91. Gemcitabine inhibits the phosphorylation of AKT 92. Previous studies by our team reported that DCK can interact with FOXO1 and that DCK is positively correlated with FOXO1 46. In this study, *UPP1* knockdown enhanced the chemosensitivity of BLCA to gemcitabine and upregulated FOXO1 and DCK. AKT overexpression mitigated the *UPP1* knockdown-induced increase in gemcitabine sensitivity by restoring the expression of DCK and FOXO1. These results suggest that *UPP1* deficiency promotes gemcitabine sensitivity through the AKT/FOXO1/DCK pathway.

However, this study has some limitations. This study experimentally verified that UPP1 overexpression promoted the phosphorylation of AKT at dual sites and identified a crucial AKT-binding site (Arg94) in UPP1 at the C-terminus. However, the specific binding site in the C-terminus of AKT for UPP1 was not examined in detail. Furthermore, the human AKT protein has three isoforms (AKT1, AKT2, and AKT3). Each isoform has a distinct subcellular localization with a specific biological function. This study did not examine the specific AKT isoform regulated by UPP1. In addition, we have provided a limited picture of UPP1 protein expression in bladder cancer by tissue microarray, and larger sample sizes are still needed to detect the generalization of UPP1 expression in bladder cancer. Tissue microarrays provide minimal epidemiological information, which makes it challenging to learn whether bladder cancer patients with specific exposure characteristics are strongly associated with UPP1 expression. Last, the expression level of UPP1 in bladder cancer cell lines was inconsistent with the malignancy of bladder cancer reported previously 93. We believe this may be related to the following reasons: first, we only detected the expression of UPP1 at the mRNA and protein levels in six bladder cancer cell lines, which is a small sample size; second, different expression patterns are widespread in various bladder cancer cell lines. For example, many oncogenes whose expression levels in bladder cancer cell lines do not correspond to the malignancy of the cell lines 94, 95. More efforts are needed to explore the biological function of UPP1 in more BLCA cell lines. These limitations will be addressed in future studies.

In conclusion, *UPP1*, an essential oncogene, promotes tumorigenesis and development via the AKT signaling pathway.

## Supplementary Material

Supplementary figures and tables.

## Abbreviations

AOD: average optical density
BCA: bicinchoninic acid
BLCA: bladder cancer
CDK2: cyclin-dependent kinase 2
co-IP: co-immunoprecipitation
DAB: diaminobenzidine
DCK: deoxycytidine kinase
FBS: fetal bovine serum
GEPIA: Gene Expression Profiling Interactive Analysis
GFP: green fluorescent protein
GLS1: glutaminase 1
GSEA: gene set enrichment analysis
GSVA: gene set variation analysis
H&E: Hematoxylin and eosin
HIS: hue-saturation-intensity
IHC: immunohistochemistry
IOD: integral optical density
MIBC: muscle-invasive bladder cancer
MTT: methyl thiazolyl tetrazolium
NMIBC: non-muscle-invasive bladder cancer
OS: overall survival
PBS: phosphate-buffered saline
PH: protein homology
PTEN: phosphatase and tensin homolog
PIP3: phosphatidylinositol 3,4,5-triphosphate
PIP2: phosphatidylinositol 4,5-bisphosphate
PI3K: phosphoinositide 3-kinase
qRT-PCR: quantitative reverse transcription PCR
ROC: receiver operating characteristic
ROS: reactive oxygen species
RT: room temperature
siRNAs: short-interfering RNAs
SDS: sodium dodecyl sulfate
TCGA: The Cancer Genome Atlas
UPP1: uridine phosphorylase 1
WT: wild-type
