# Supplementary material for: UPP1 enhances bladder cancer progression and gemcitabine resistance through AKT
Source: Int J Biol Sci. 2024 Jan 27;20(4):1389–409. doi: 10.7150/ijbs.83774 (PMC10878145; doi:10.7150/ijbs.83774)
Supplement: Supplementary file 1 — Supplementary figures and tables. [file ijbsv20p1389s1.pdf]

Supplementary Materials

UPP1 enhances bladder cancer progression and gemcitabine resistance through AKT

Supplementary Figures: Pages 2-23

Supplementary Tables: Pages 24-29

Supplementary Figures S1-S15

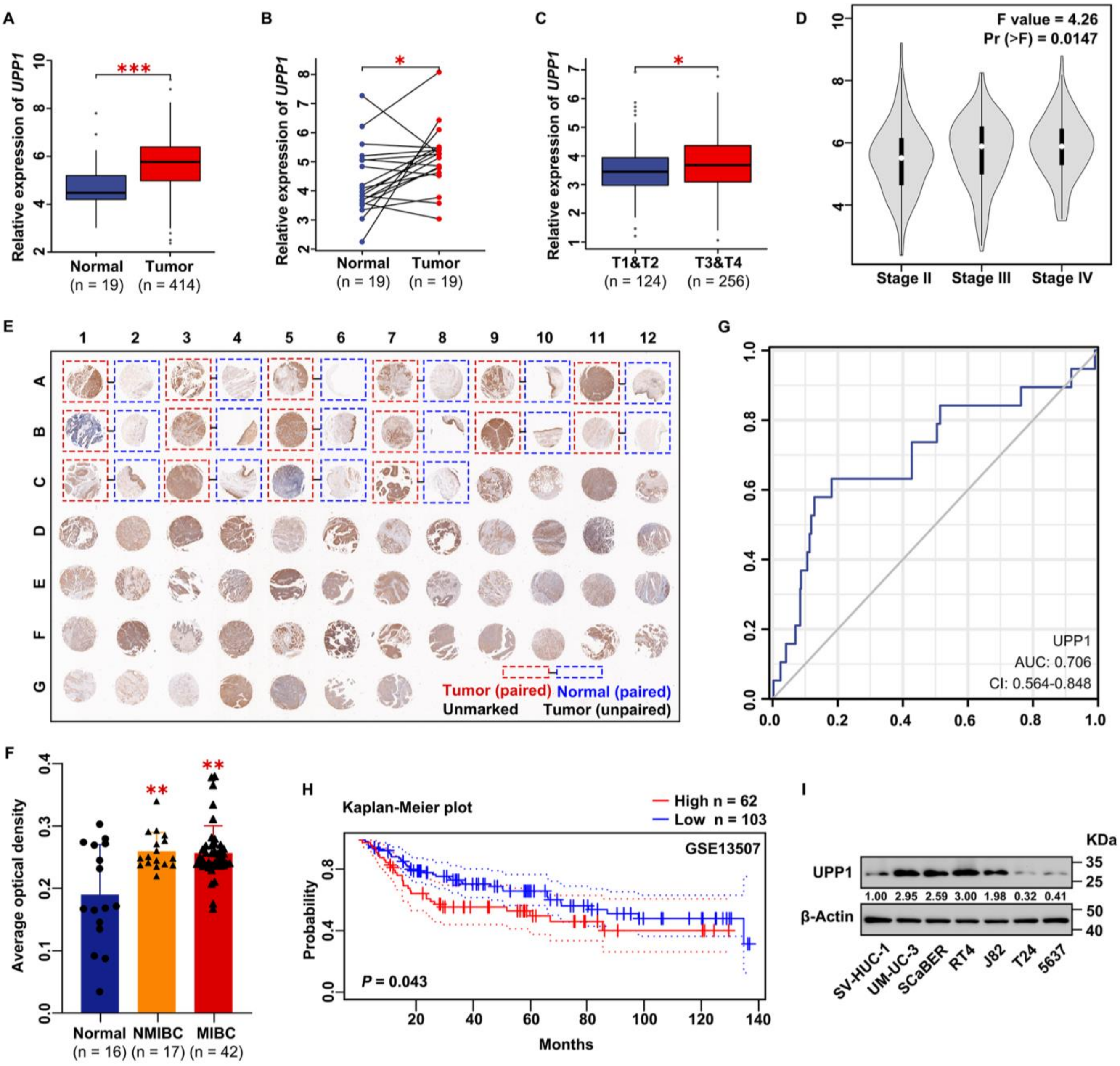

Supplementary Figure S1. UPP1 is highly expressed and positively correlated with prognosis in BLCA. Relative expression of *UPP1* in unpaired **(A)** and paired **(B)** adjacent tissues and BLCA tissues based on TCGA databases. **(C)** Relative expression of *UPP1* in unpaired T1 & T2 BLCA and T3 & T4 BLCA based on TCGA databases. **(D)** Relative expression of *UPP1* in different stages of BLCA provided by GEPIA. **(E)** Holistic representation of UPP1 immunohistochemical staining of the tissue microarray. **(F)** Relative expression of normal tissue, NMBIC, and MIBC in the tissue microarray. **(G)** ROC curve analysis of UPP1 in normal and tumor samples. AUC score indicates that UPP1 has a certain accuracy in predicting the prognosis of BLCA. **(H)** Kaplan-Meier survival plot based on the GSE13507 dataset presented by the PrognoScan database. **(I)** The protein level of UPP1 in BLCA cell lines was evaluated by immunoblotting. *: $p < 0.05$; **: $p < 0.01$; ***: $p < 0.001$; ns: not significant ($p > 0.05$).

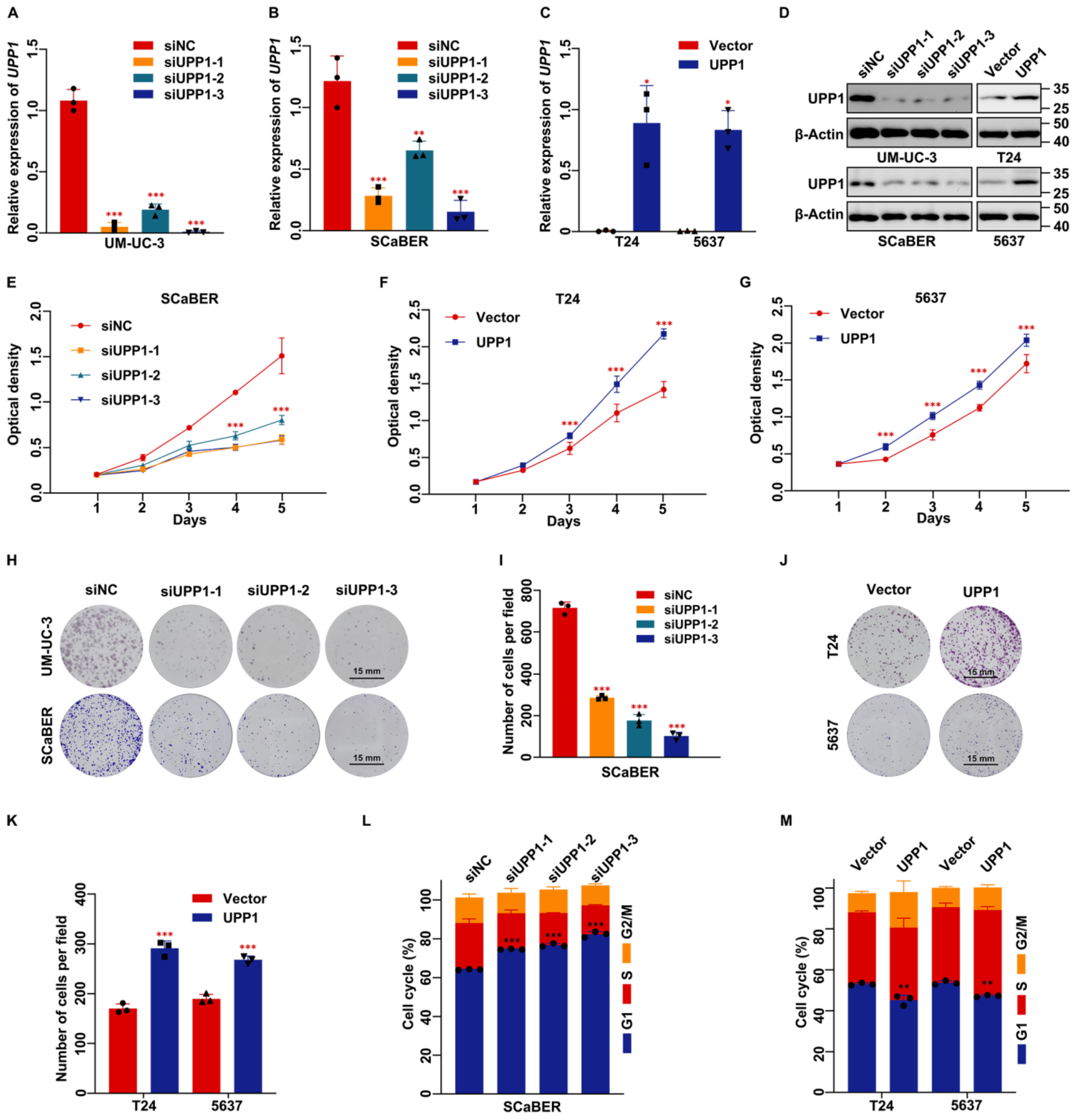

Supplementary Figure S2. ***UPP1*** **deficiency inhibited the proliferation of BLCA cells *in vitro*.**

UPP1 knockdown efficiency of 3 siRNAs in UM-UC-3 **(A)** and SCaBER **(B)** cells and **(C)** UPP1 overexpression efficiency in T24 and 5637 cells evaluated by qRT-PCR. **(D)** *UPP1* knockdown efficiency of 3 siRNAs in UM-UC-3 and SCaBER cells and UPP1 overexpression efficiency in T24 and 5637 cells evaluated by immunoblotting. **(E)** MTT assay shows that *UPP1* deficiency inhibits the proliferation of SCaBER cells. MTT assay of UPP1 overexpression in T24 **(F)** and 5637 **(G)** cells. The clonogenic assay **(H)** and the statistical chart **(I)** of downregulating *UPP1* in UM-UC-3 and SCaBER cells. Clonogenic assay **(J)** and the statistical chart **(K)** of UPP1 overexpression in T24 and 5637 cells. Statistical chart of the cell cycle

analysis of *UPP1* deficiency **(L)** and UPP1 overexpression **(M)** in multiple BLCA cell lines. *: $p < 0.05$; **: $p < 0.01$; ***: $p < 0.001$; ns: not significant ($p > 0.05$).

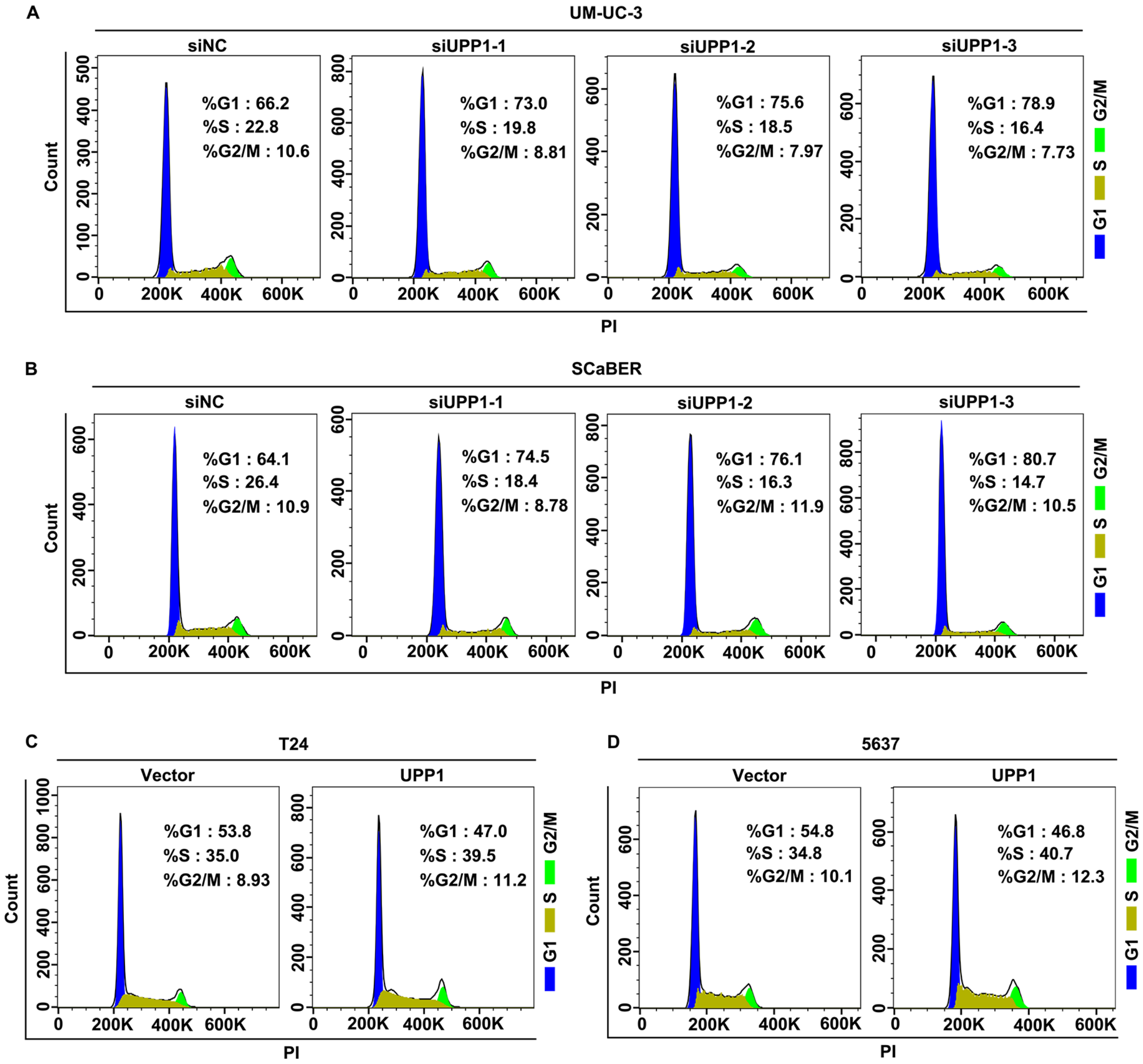

Supplementary Figure S3. UPP1 can promote cell cycle transition in BLCA cells.

Flow cytometry analysis indicates that *UPP1* deficiency can promote cell cycle arrest in **(A)** UM-UC-3 and **(B)** SCaBER cells, and overexpressing UPP1 can accelerate cell cycle transition in **(C)** T24 and **(D)** 5637 cells.

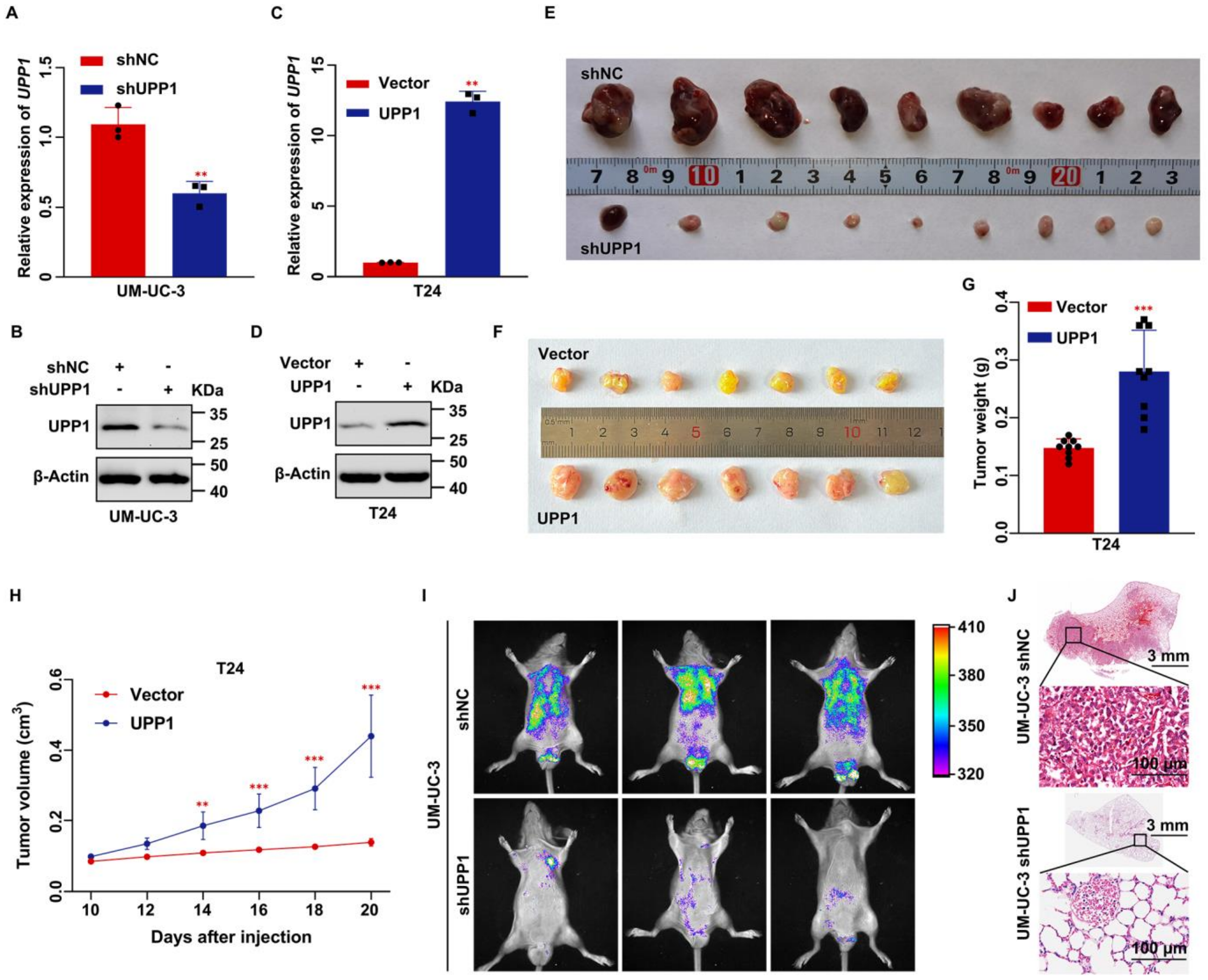

Supplementary Figure S4. *UPP1* deficiency inhibited BLCA cell proliferation and migration *in vivo.* Knockdown efficiency of the stable knockdown *UPP1* cell line UM-UC-3 shUPP1 compared with UM-UC-3 shNC evaluated by qRT-PCR **(A)** and immunoblotting **(B)**. The overexpression efficiency of the stably overexpressing UPP1 cell line of Vector-transfected and UPP1-transfected T24 cells was evaluated by qRT-PCR **(C)** and immunoblotting **(D)**. **(E)** The volume of subcutaneous xenograft tumors of shNC-transfected and shUPP1-transfected UM-UC-3 cells. The volume **(F)**, weight **(G),** and growth curve **(H)** of subcutaneous xenograft tumors of Vector-transfected and UPP1-transfected T24 cells. Fluorescence intensity **(I)** and H&E staining **(J)** of the lung from the BALB/c nude mouse metastasis model injected with shNC-transfected and shUPP1-transfected UM-UC-3 cells. *: $p < 0.05$; **: $p < 0.01$; ***: $p < 0.001$; ns: not significant ($p > 0.05$).

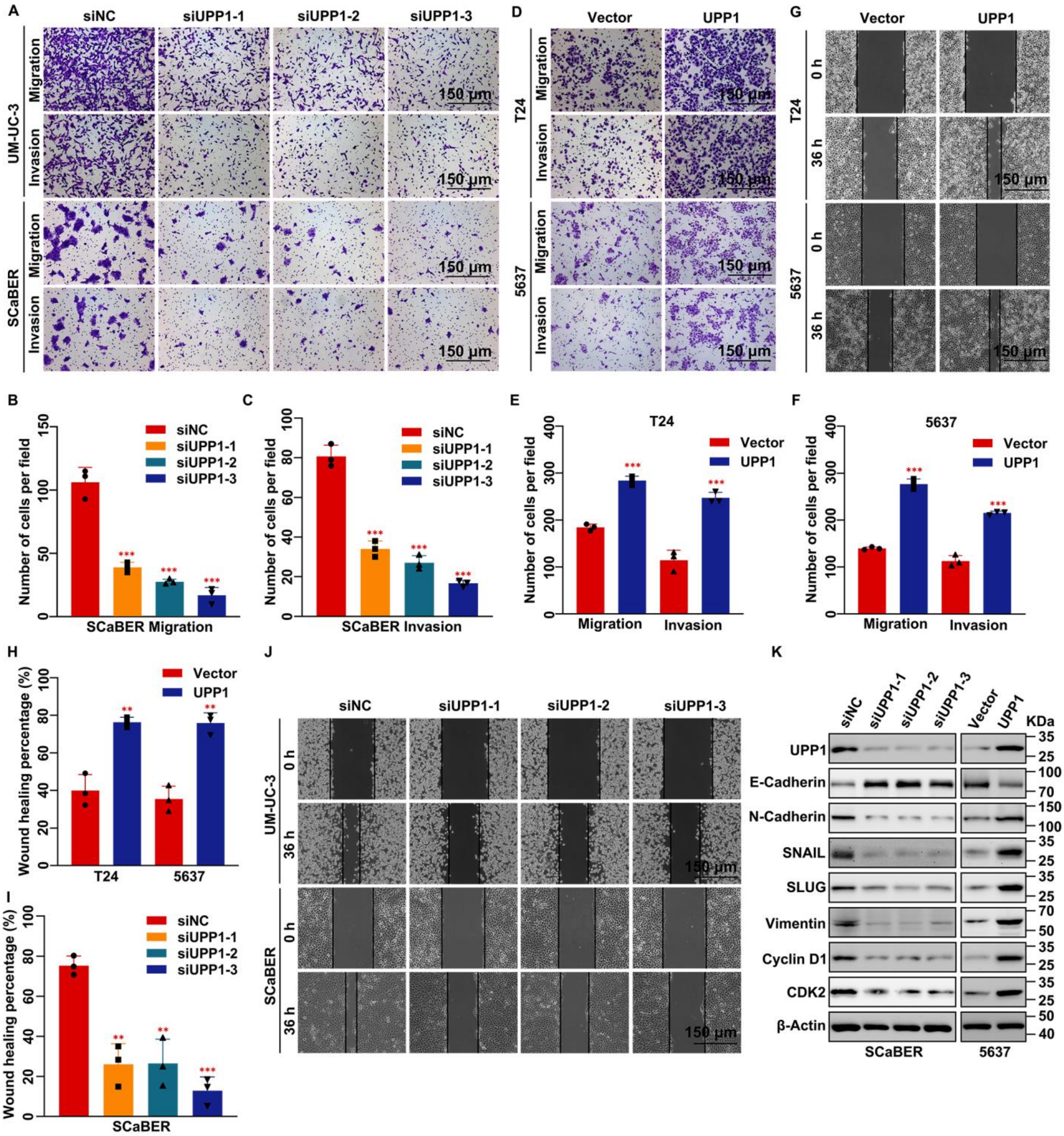

Supplementary Figure S5. Downregulating *UPP1* can suppress migration and invasion in BLCA cells. **(A-C)** Transwell assay and statistical chart of *UPP1* deficiency in UM-UC-3 and SCaBER cells. **(D-F)** UPP1 overexpression can promote migration and invasion in T24 and 5637 cells. **(G-J)** Wound healing assay and statistical chart of UPP1 overexpression and *UPP1* downregulation in multiple BLCA cell lines. **(K)** Immunoblotting indicates that EMT- and cell cycle-related proteins are positively associated with the expression of UPP1. *: $p < 0.05$; **: $p < 0.01$; ***: $p < 0.001$; ns: not significant ($p > 0.05$).

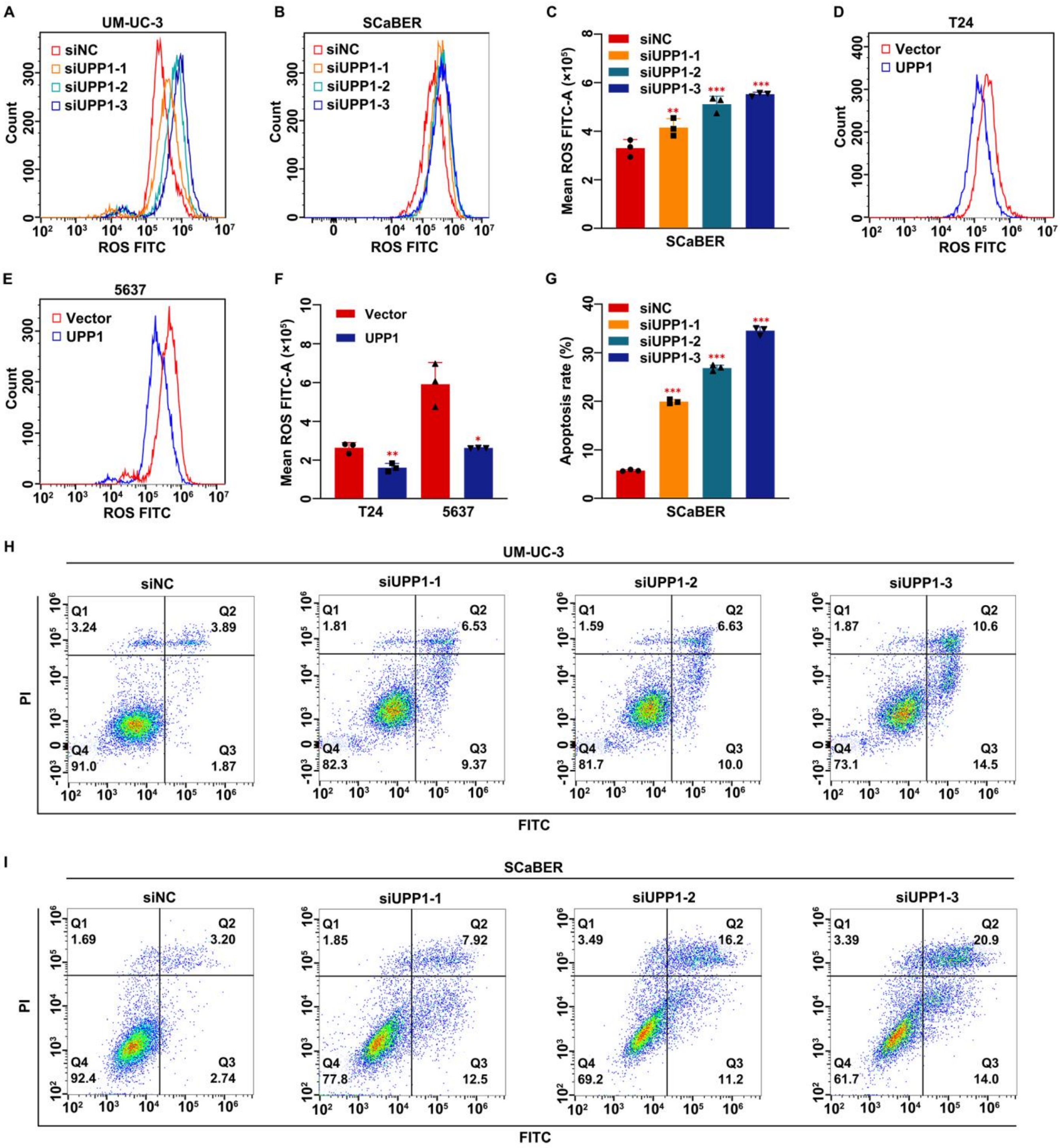

Supplementary Figure S6. Overexpressing UPP1 can lower the level of cellular ROS and inhibit apoptosis.

(A-F) Flow cytometry assay and statistical chart of *UPP1* deficiency and UPP1 overexpression to detect cellular ROS levels in multiple BLCA cells. **(G-I)** Statistical chart and flow cytometry analysis of the apoptosis rate of *UPP1*-deficient in SCaBER and UM-UC-3 cells. *: $p < 0.05$; **: $p < 0.01$; ***: $p < 0.001$; ns: not significant ($p > 0.05$).

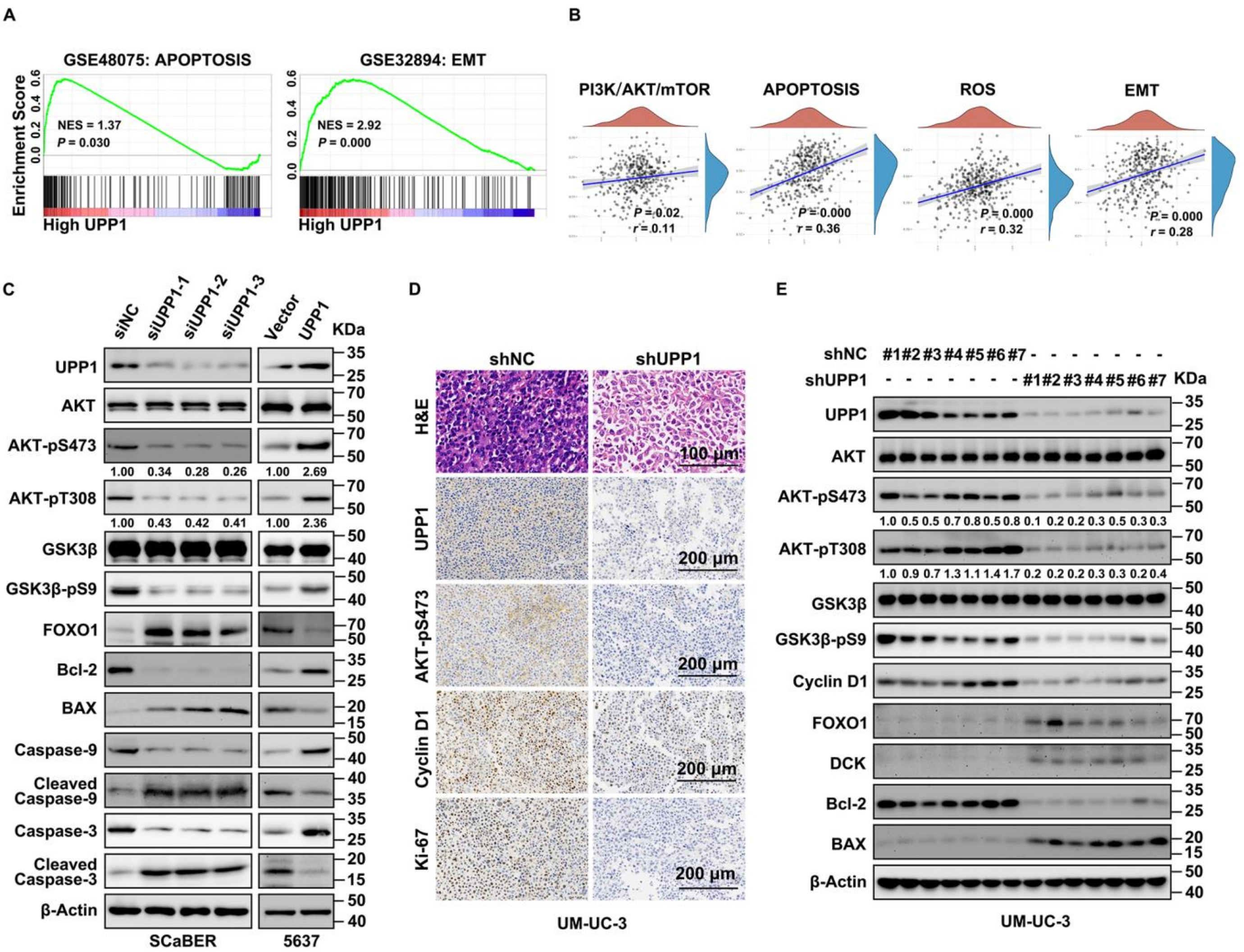

Supplementary Figure S7. *UPP1* deficiency inhibits the AKT signaling pathway *in vitro* and *in vivo*.

(A) GSEA and **(B)** GSVA of UPP1. **(C)** Immunoblotting indicated that knockdown of *UPP1* can lower AKT phosphorylation and promote apoptosis of BLCA cells *in vitro* and *in vivo*. **(D)** H&E and IHC staining of subcutaneous shNC-transfected and shUPP1-transfected UM-UC-3 cell-derived xenograft tumors. **(E)** Immunoblotting indicates that *UPP1* knockdown could inhibit AKT phosphorylation and promote apoptosis of BLCA cells *in vivo*.

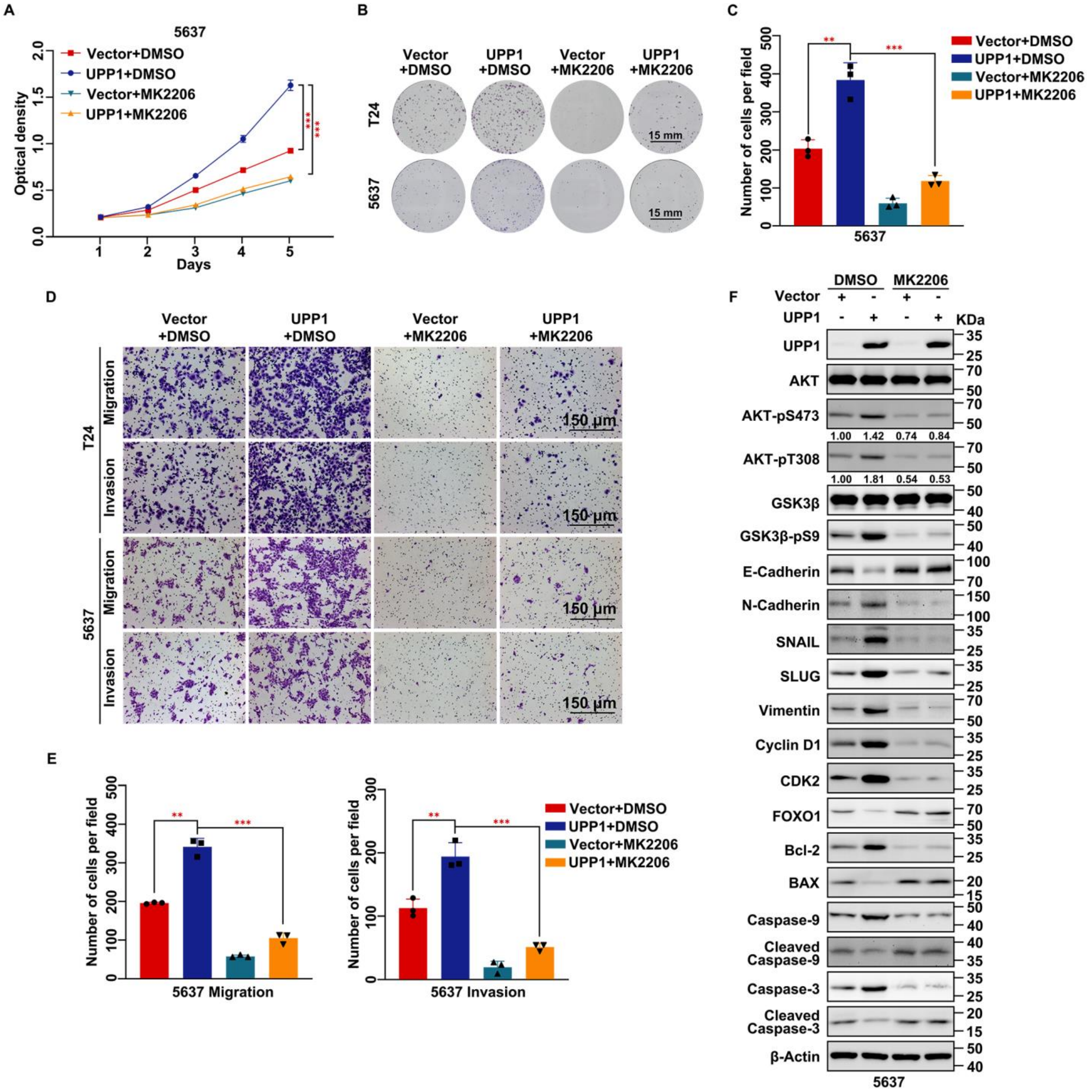

Figure S8. Suppressing AKT rescues the cancer-promoting effect of UPP1 overexpression in BLCA cells. **(A)** MTT assay results revealed that treatment with MK2206 (10 μM) suppressed the UPP1-induced upregulation of 5637 cell proliferation. **(B-C)** The colony formation assay revealed that MK2206 (10 μM) mitigated the UPP1-induced upregulation of T24 and 5637 cell proliferations. **(D–E)** The statistical chart of the transwell assay results indicates that MK2206 (10 μM) suppressed the UPP1-induced upregulation of migration and invasion in T24 and 5637 cells. **(F)** Immunoblotting analysis revealed that UPP1-induced AKT activation was mitigated upon treatment with MK2206 (10 μM). *: $p < 0.05$; **: $p < 0.01$; ***: $p < 0.001$; ns: not significant ($p > 0.05$).

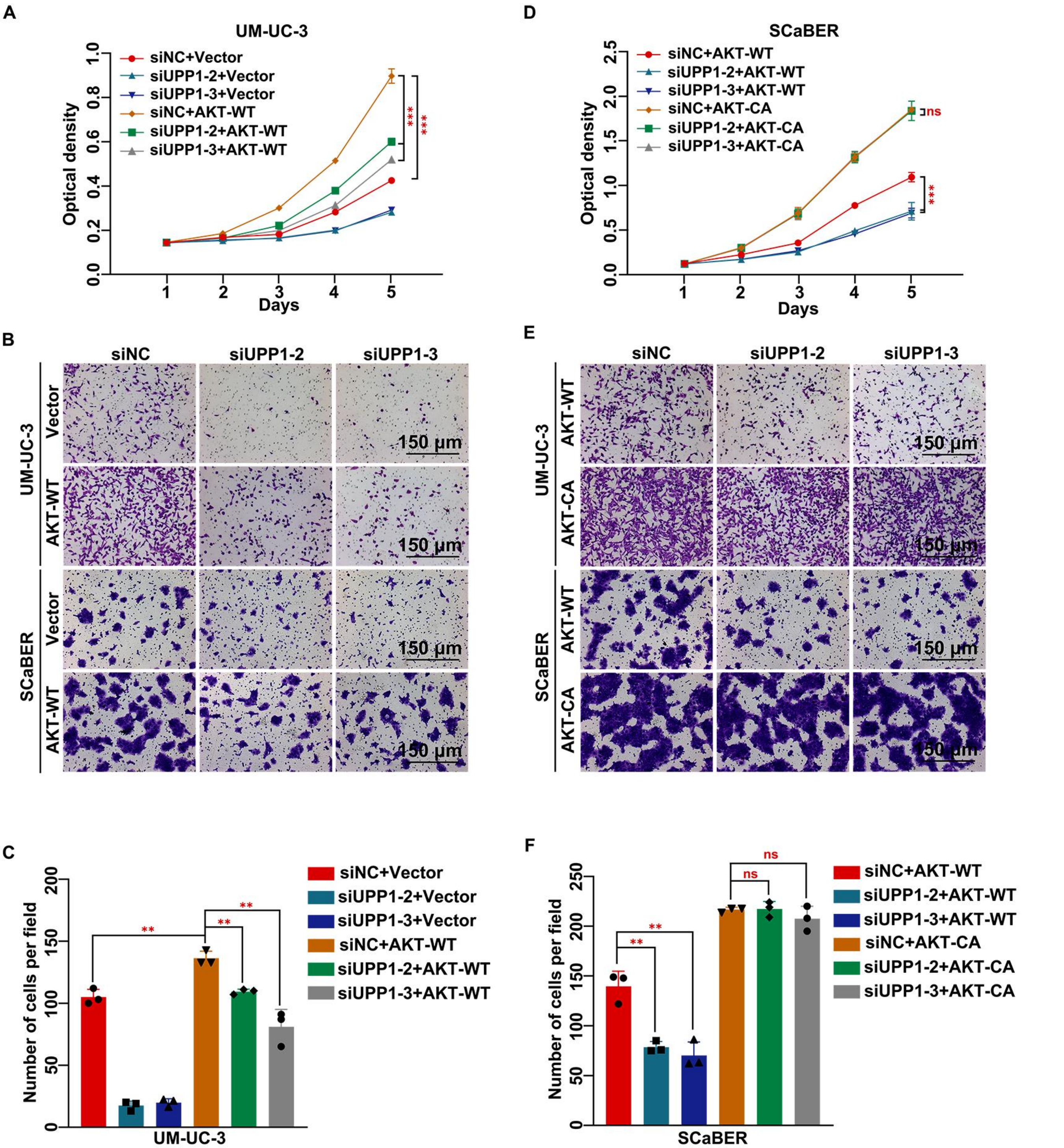

Supplementary Figure S9. AKT overexpression can rescue tumor inhibition by *UPP1* knockdown.

(A) MTT assay of *UPP1* knockdown with AKT-WT overexpression in UM-UC-3 cells. The transwell assay **(B)** and statistical chart **(C)** indicate that overexpressing AKT-WT can partially recover the migration ability of UM-UC-3 and SCaBER cells. **(D)** MTT assay indicates that the weakened proliferation from *UPP1* deficiency can be completely rescued by overexpressing AKT-CA in SCaBER cells. **(E)** Transwell assay and **(F)** statistical chart of AKT-CA overexpression in UM-UC-3 and SCaBER cells. *: $p < 0.05$; **: $p < 0.01$; ***: $p < 0.001$; ns: not significant ($p > 0.05$).

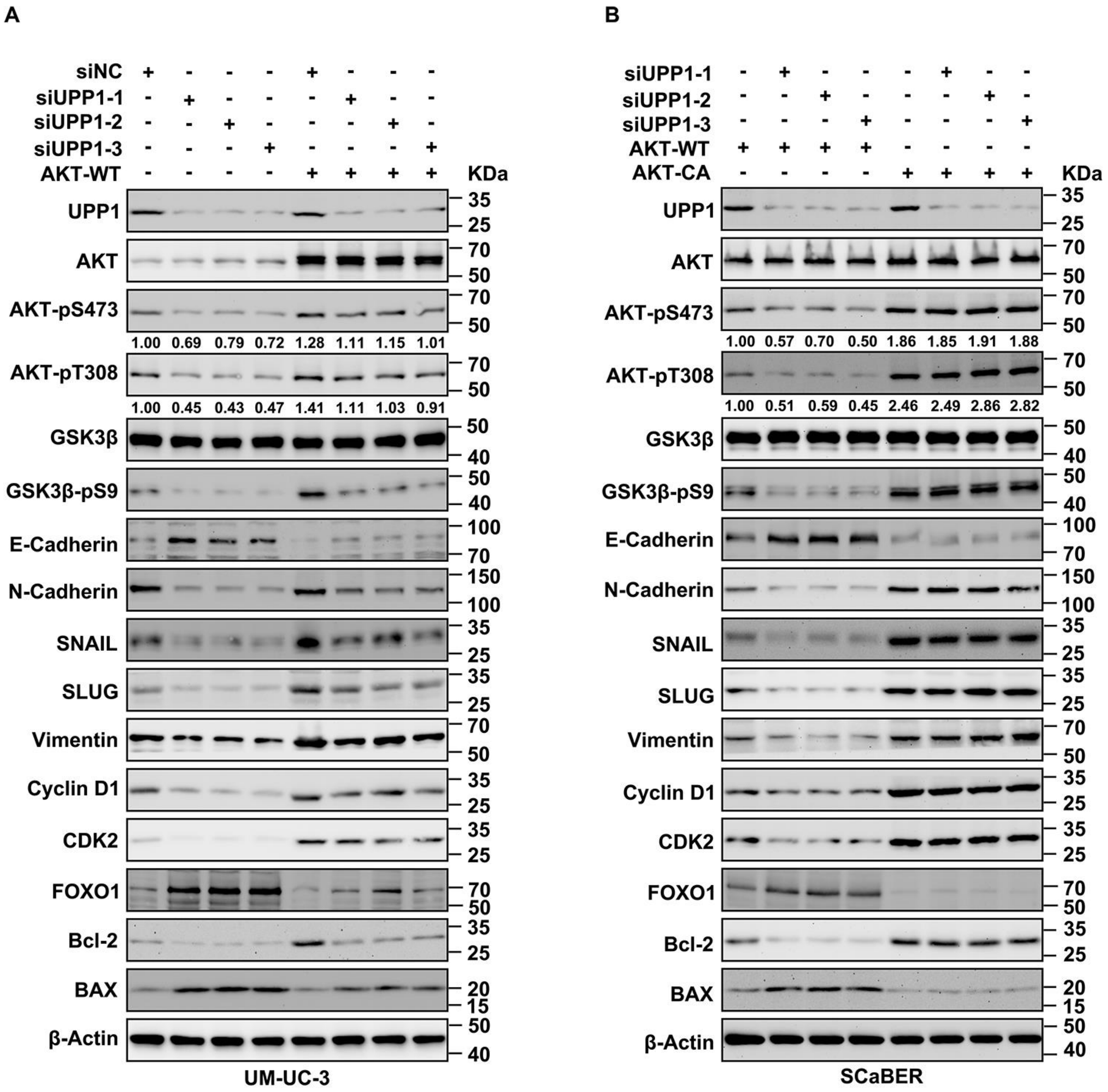

Supplementary Figure S10. Overexpressing AKT can restore the suppressive effects of *UPP1* deficiency on BLCA.

(A-B) Overexpressing AKT-CA recovered the suppressive effects produced by *UPP1* deficiency compared with AKT-WT.

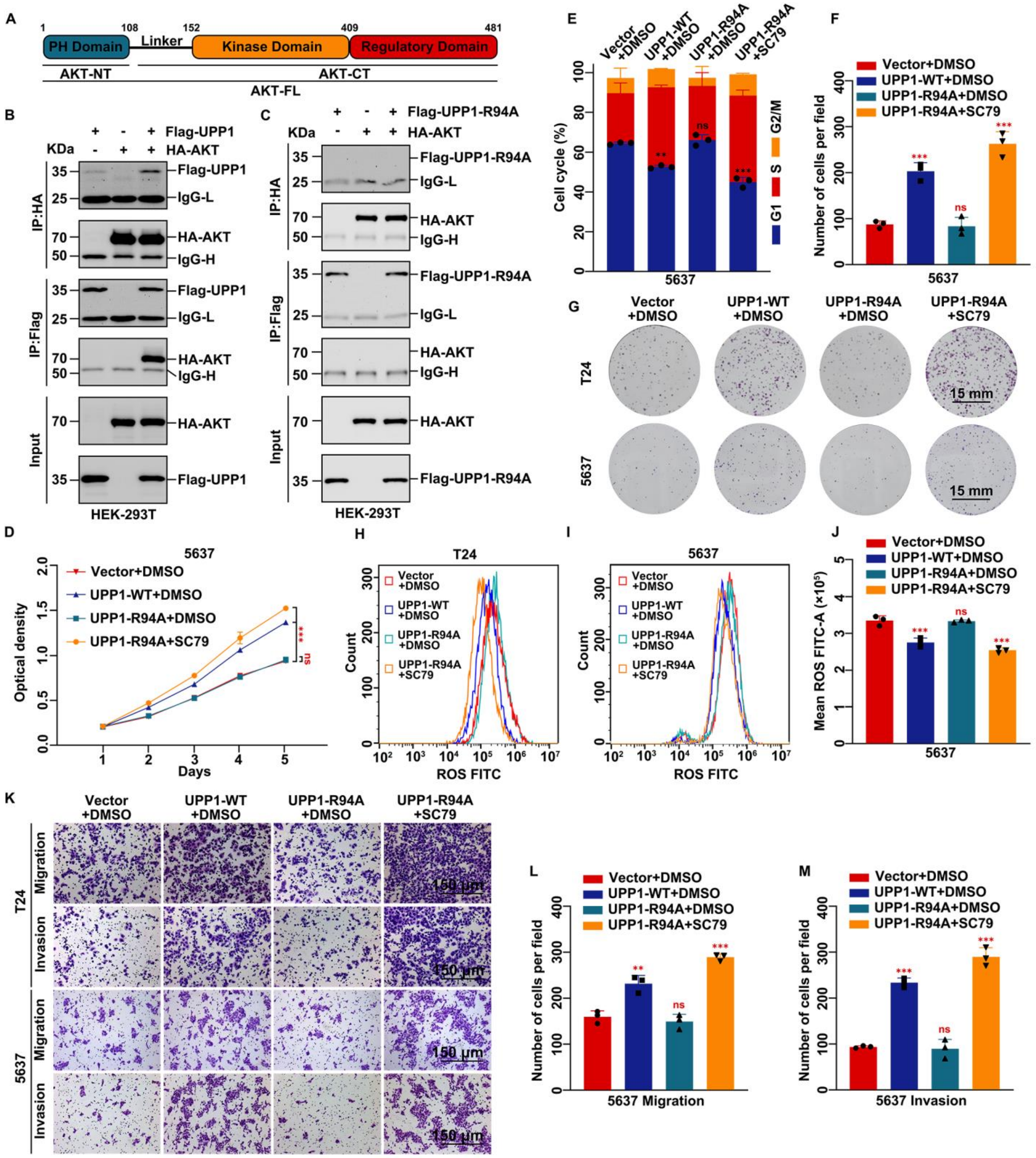

Supplementary Figure S11. UPP1-R94A cannot interact with AKT and promote BLCA malignancy.

(A) Schematic of the AKT truncated plasmid. **(B-C)** Co-IP indicates that UPP1-WT can interact with AKT, but UPP1-R94A cannot interact with AKT in HEK-293T cells. **(D)** MTT assay of 5637 cells overexpressing UPP1-R94A. **(E)** Statistical chart of the cell cycle analysis of 5637 cells overexpressing UPP1-R94A. SC79 (20 μM) significantly promotes cell cycle transition. Clonogenic assay **(F)** and statistical chart **(G)** of UPP1-R94A overexpression in T24 and 5637 cells. SC79 (20 μM) rebuilds the promotion effects. **(H-J)** Overexpressing UPP1-R94A did not change the cellular ROS level in T24 and 5637 cells, but SC79 (20 μM)

significantly lowered the cellular ROS level. **(K-M)** Transwell assay and statistical chart of UPP1-R94A overexpression in T24 and 5637 cells. SC79 (20 μM) can significantly promote metastasis of BLCA cells. *: $p < 0.05$; **: $p < 0.01$; ***: $p < 0.001$; ns: not significant ($p > 0.05$).

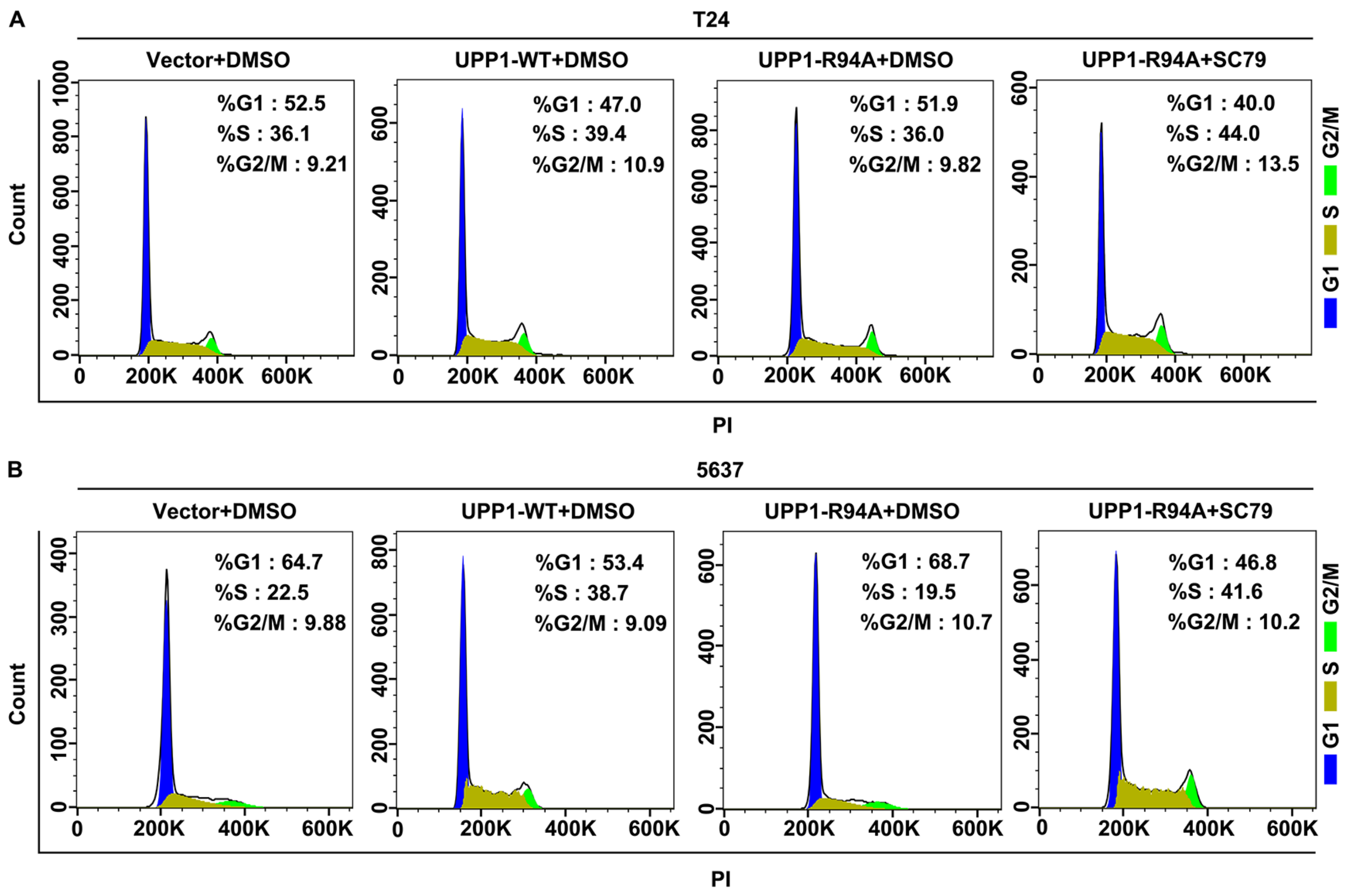

Supplementary Figure S12. Overexpressing UPP1-R94A does not promote cell cycle transition in BLCA cells.

Flow cytometry shows that overexpressing UPP1-R94A cannot accelerate the cell cycle in **(A)** T24 and **(B)** 5637 cells, whereas SC79 (20 μM) fully activates this process.

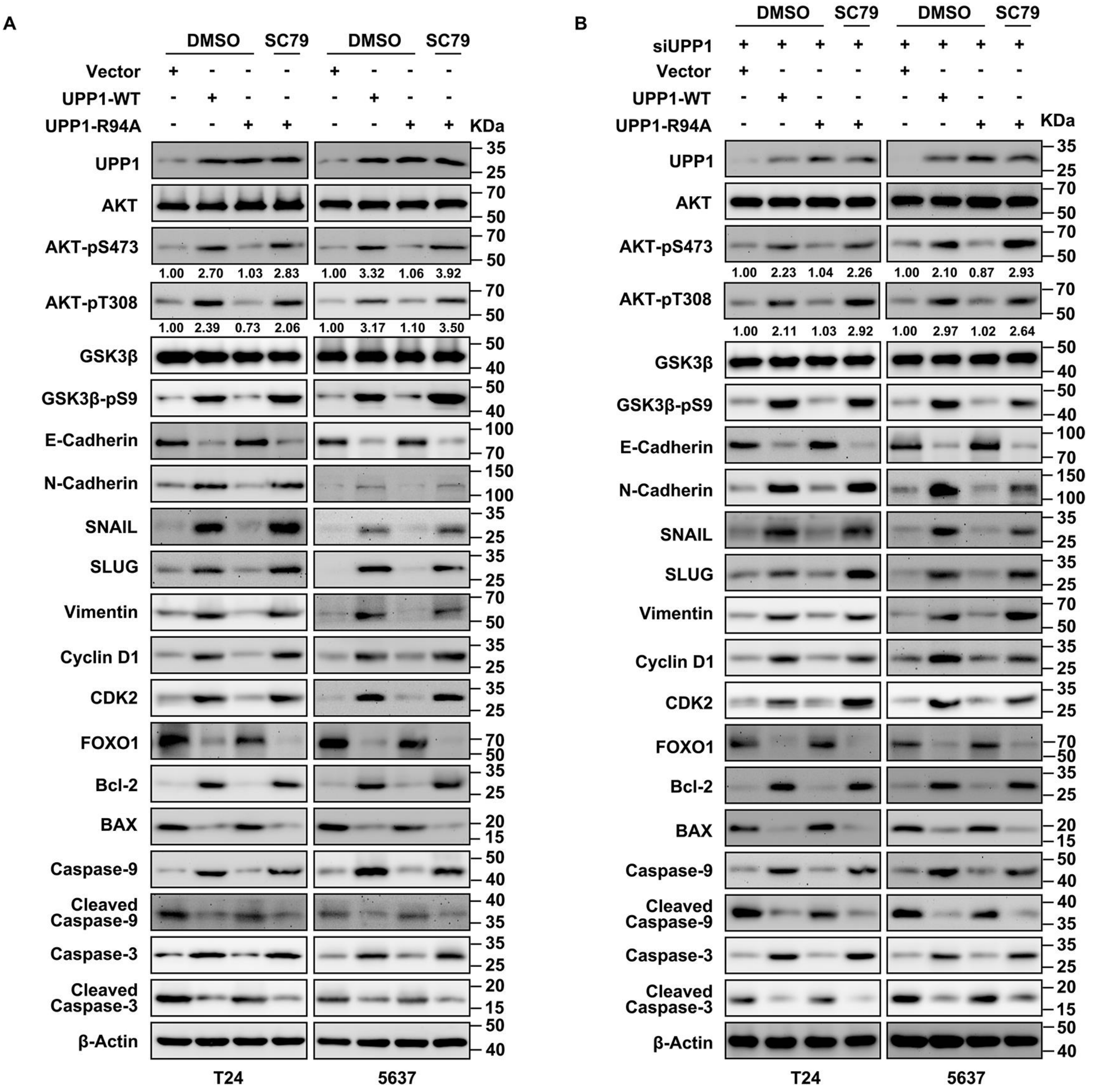

Supplementary Figure S13. Overexpressing UPP1-R94A fails to promote the activation of the AKT signaling pathway.

(A-B) Immunoblotting indicates that UPP1-R94A cannot activate the AKT signaling pathway regardless of whether endogenous UPP1 is removed in T24 and 5637 cells.

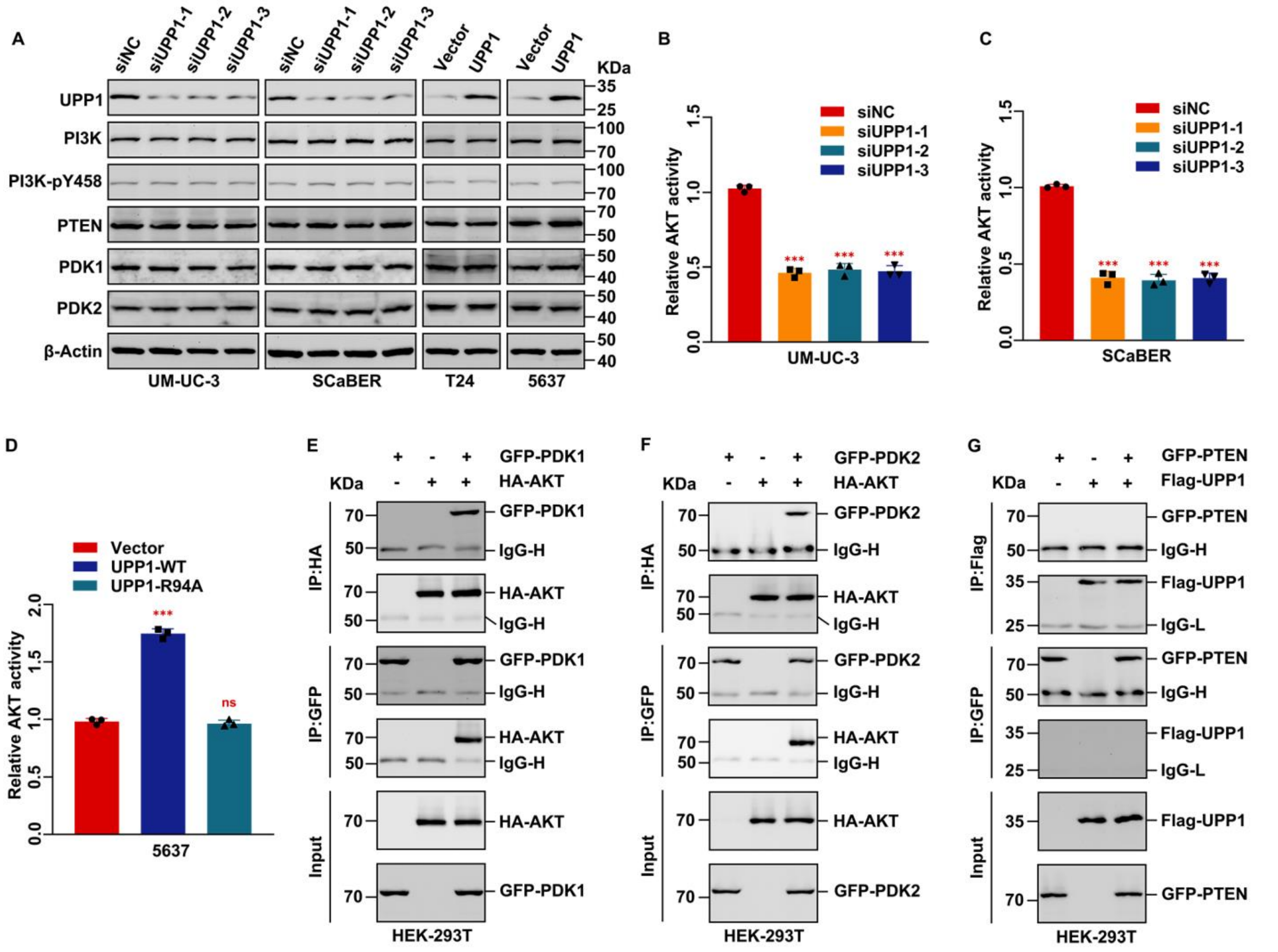

Supplementary Figure S14. UPP1 promotes AKT activation.

(A) Immunoblotting shows that *UPP1* deficiency or UPP1 overexpression cannot change the expression of PI3K, PI3K-pY458, PTEN, PDK1, and PDK2 in UM-UC-3, SCaBER, T24, and 5637 cells. **(B-C)** UPP1 deficiency inhibits AKT activity in UM-UC-3 and SCaBER cells. **(D)** Relative AKT activity after overexpressing UPP1-WT or UPP1-R94A in 5637 cells. **(E-F)** Exogenous co-IP indicates that AKT interacts with PDK1 and PDK2 in HEK-293T cells. **(G)** Exogenous co-IP shows that UPP1 does not interact with PTEN in HEK-293T cells. *: $p < 0.05$; **: $p < 0.01$; ***: $p < 0.001$; ns: not significant ($p > 0.05$).

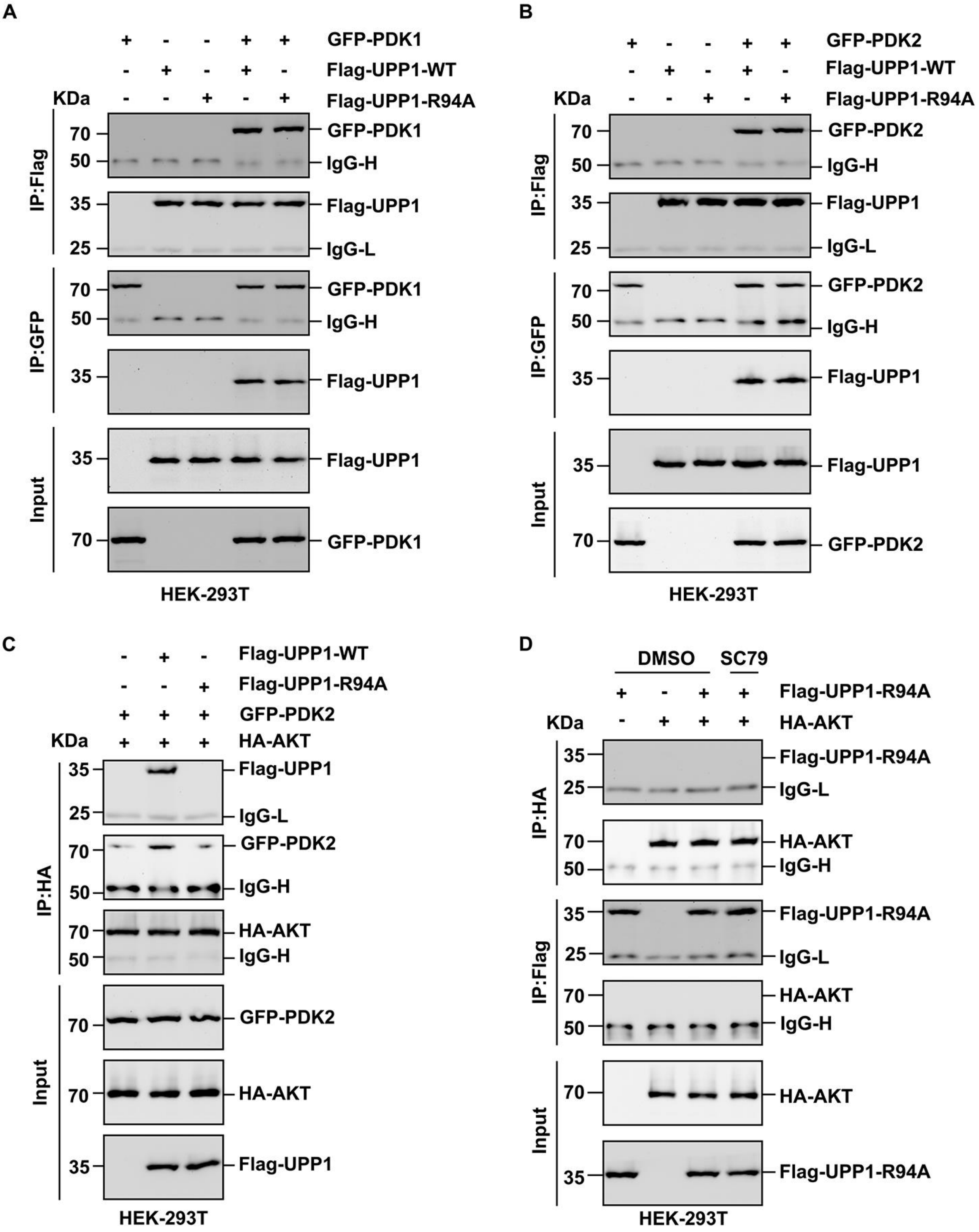

Supplementary Figure S15. UPP1 facilitates AKT phosphorylation by strengthening the binding of AKT to PDK1 and PDK2 in HEK-293T cells.

(A-B) Exogenous co-IP shows that UPP1 interacts with PDK1 and PDK2 in HEK-293T cells. **(C)** Quantitative co-IP shows that UPP1 promotes the binding of AKT to PDK2 in HEK-293T cells. **(D)** Exogenous co-IP demonstrates that UPP1-R94A does not interact with AKT regardless of whether SC79 is implemented in HEK-293T cells.

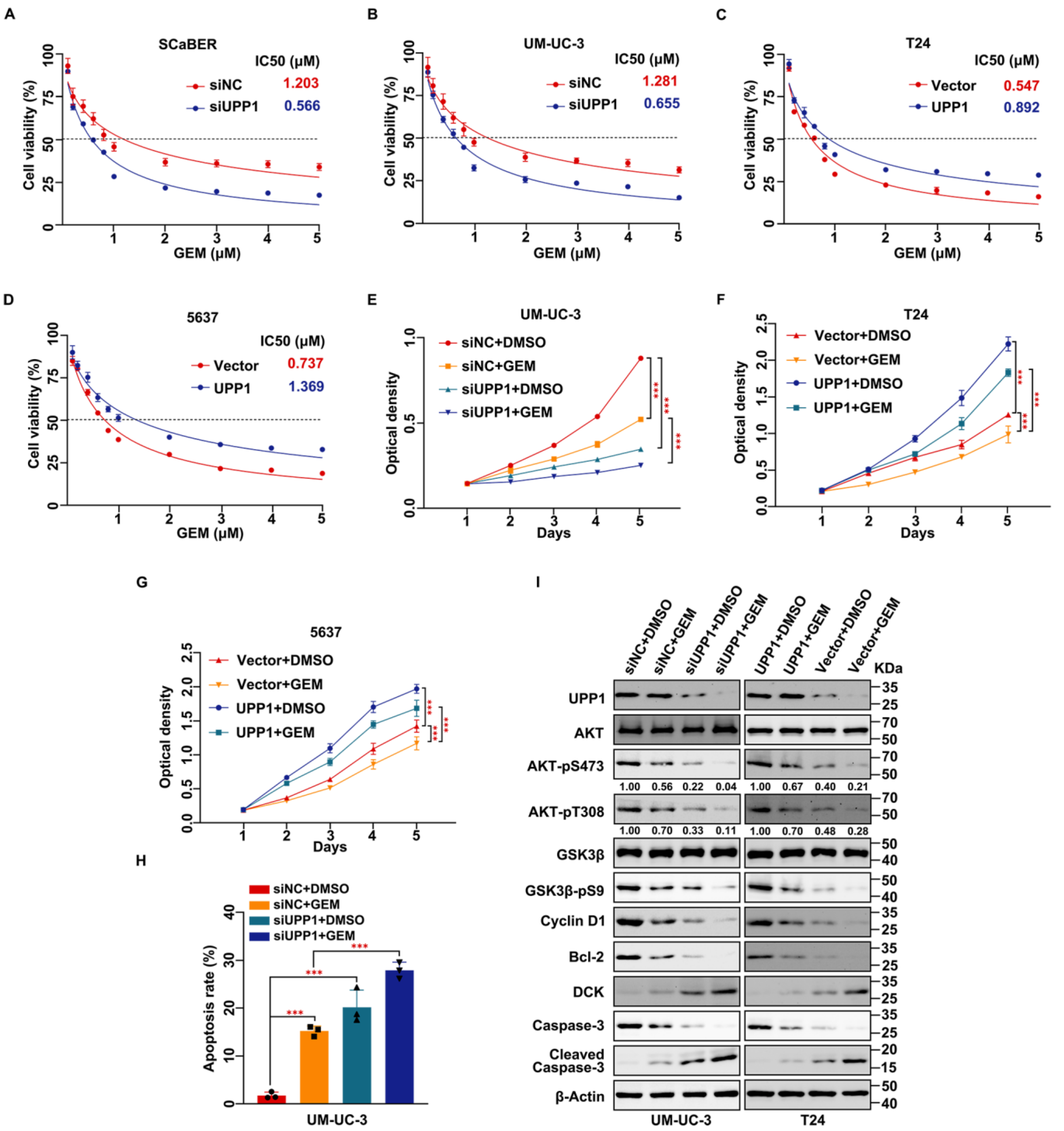

Supplementary Figure S16. ***UPP1*** **deficiency promotes gemcitabine chemosensitivity in BLCA cells by downregulating the AKT signaling pathway *in vitro*.**

(A-D) IC50 of gemcitabine in multiple BLCA cell lines. **(E)** MTT assay indicates that *UPP1* deficiency can promote gemcitabine chemosensitivity in UM-UC-3 cells, and overexpressing UPP1 can facilitate gemcitabine resistance in **(F)** T24 and **(G)** 5637 cells. **(H)** *UPP1* deficiency with gemcitabine significantly increased the apoptosis rate in UM-UC-3 cells. **(I)** Immunoblotting indicates that downregulating *UPP1* can deepen the inhibition of AKT phosphorylation by gemcitabine, and overexpressing UPP1 can partially reverse these inhibitory effects. *: $p < 0.05$; **: $p < 0.01$; ***: $p < 0.001$; ns: not significant ($p > 0.05$).

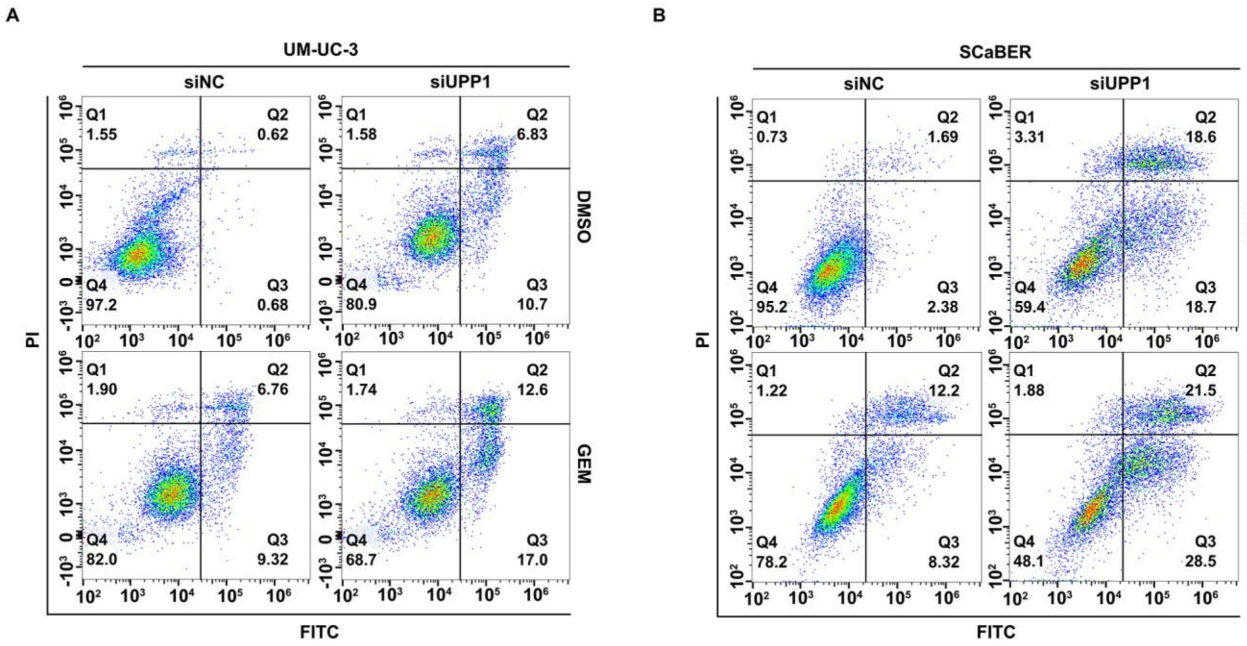

Supplementary Figure S17. ***UPP1*** **deficiency strengthens gemcitabine chemosensitivity in BLCA cells by promoting apoptosis** ***in vitro*****.**

(A-B) Flow cytometry analysis of the apoptosis rate with *UPP1* deficiency in UM-UC-3 and SCaBER cells.

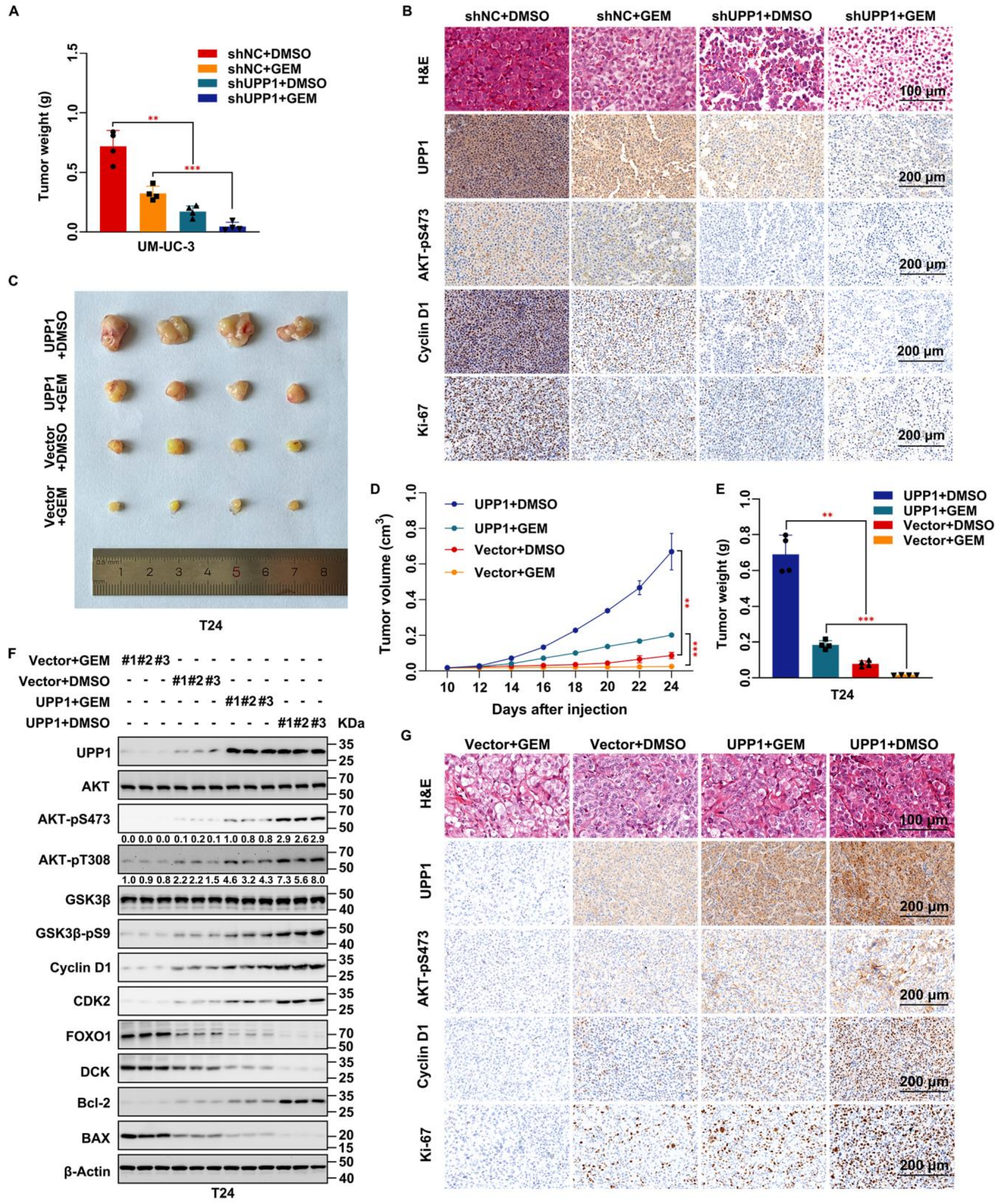

Supplementary Figure S18. UPP1 promotes the gemcitabine resistance of bladder cancer (BLCA) cells by activating the AKT signaling pathway *in vivo*.

The weight **(A)** of tumors derived from shNC-transfected or shUPP1-transfected UM-UC-3 cells combined with gemcitabine treatment. **(B)** H&E and IHC staining of subcutaneous shNC-transfected and shUPP1-transfected UM-UC-3 cell-derived xenograft tumors combined with gemcitabine treatment. The volume **(C)**, weight **(D)** and growth curve **(E)** of subcutaneous xenograft tumors of Vector-transfected and UPP1-

transfected T24 cells combined with gemcitabine implementation. Immunoblot **(F)**, H&E and IHC **(G)** staining of subcutaneous Vector-transfected and UPP1-transfected T24 cell-derived xenograft tumors combined with gemcitabine treatment indicated that UPP1 overexpression could promote AKT phosphorylation and inhibit apoptosis of BLCA cells *in vivo*. *: $p < 0.05$; **: $p < 0.01$; ***: $p < 0.001$; ns: not significant ($p > 0.05$).

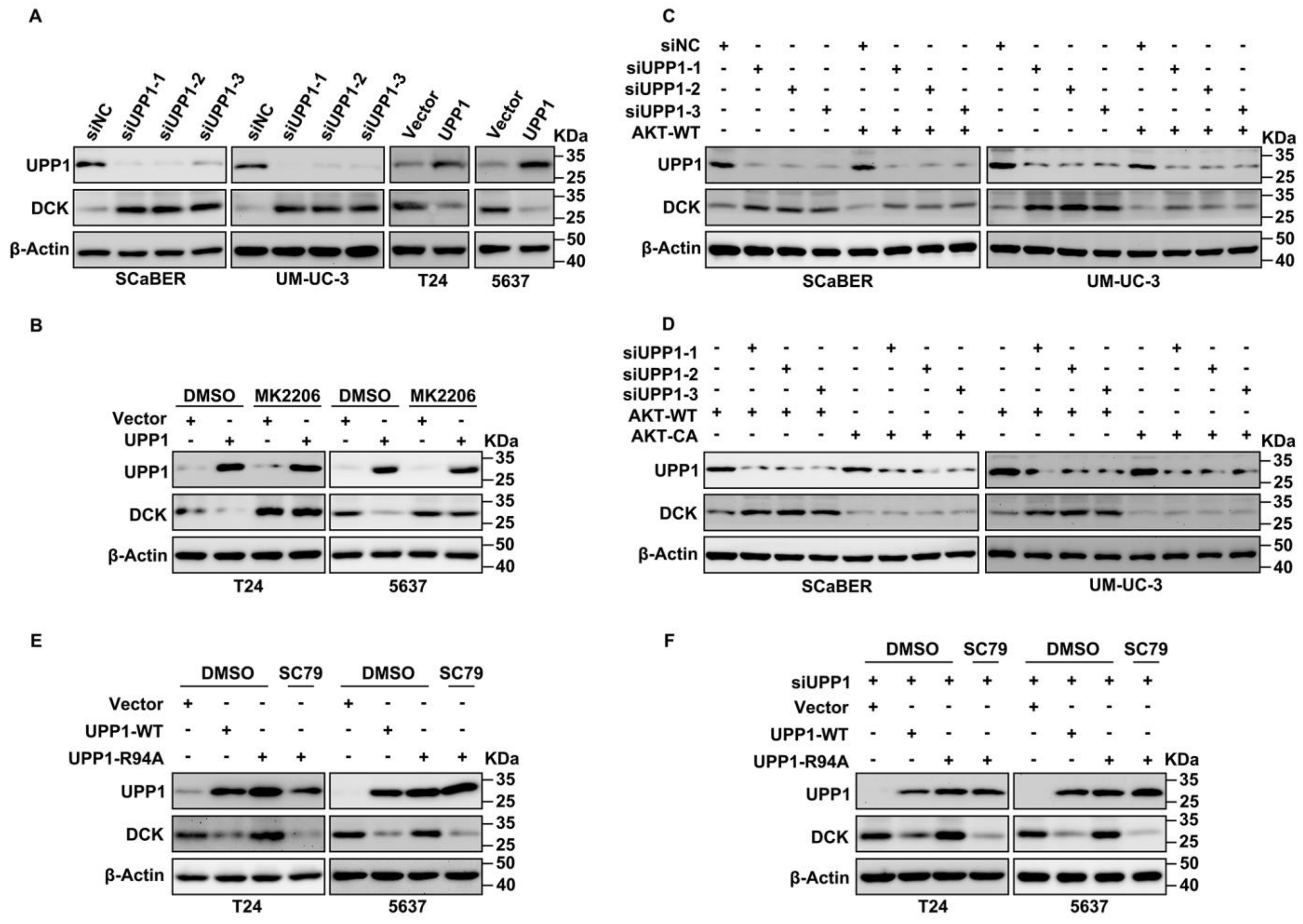

Supplementary Figure S19. UPP1 regulates gemcitabine chemosensitivity in BLCA cells by regulating the expression of DCK.

(A-D) *UPP1* deficiency can facilitate the expression of DCK, and overexpressing AKT can downregulate the level of DCK. **(E-F)** Overexpressing UPP1-R94A does not inhibit the level of DCK, regardless of whether endogenous UPP1 is eliminated.

Supplementary Tables S1-S5

Supplementary Table S1. Details of antibodies used in this study.

Antibody	Commercial sources	Catalog No.
UPP1 (for Immunoblot)	Abcam	ab128854
UPP1 (for IHC)	Proteintech	14186-1-AP
β-Actin	Santa Cruz	sc-47778
N-Cadherin	Cell Signaling Technology	13116S
E-Cadherin	Abcam	ab76055
Vimentin	Cell Signaling Technology	5741S
SLUG	Cell Signaling Technology	9585S
SNAIL	Cell Signaling Technology	3879S
AKT	Cell Signaling Technology	4691L
AKT-pS473	Cell Signaling Technology	4060L
AKT-pT308	Cell Signaling Technology	9275L
GSK3β	Cell Signaling Technology	12456S
GSK3β-pS9	Cell Signaling Technology	5558S
FOXO1A	Abcam	ab52857
Cyclin D1	Abcam	ab134175
CDK2	Abcam	ab32147
Bcl-2	Cell Signaling Technology	15071S
BAX	Cell Signaling Technology	2772S
Caspase-3	Proteintech	19677-1-AP
Cleaved Caspase-3	Cell Signaling Technology	9664S
DCK	Proteintech	17758-1-AP
Caspase-9	Cell Signaling Technology	9508S
PI3K	Cell Signaling Technology	4257
PI3K-pY458	Cell Signaling Technology	4228S
PTEN	Cell Signaling Technology	9559S
PDK1	Proteintech	18262-1-AP
PDK2	Proteintech	15647-1-AP
FLAG-Tag	Sigma	F1804
HA-Tag	OriGene	TA180128
His-Tag	Proteintech	66005-1-Ig
GFP-Tag	Santa Cruz	sc-9996
GST-Tag	Proteintech	10000-0-AP
Ki-67	Abcam	ab16667

*For immunoblotting, the dilution of the above antibodies is 1:1000.

*For IHC staining, the dilution of UPP1, AKT-pS473, Cyclin D1 and Ki-67 antibodies is 1:200.

*For immunofluorescence assay, the dilution of HA and Flag antibodies is 1:100.

Supplementary Table S2. The sequences of siRNA, shRNA, and UPP1 overexpression lentivirus used in this study.

**siRNA**	**Sequences**
siNC	5’-UUCUCCGAACGUGUCACGUTT-3’
siUPP1-1	5’-GCUGAAAGUCACAAUGAUUTT-3’
siUPP1-2	5’-CCCAGCCUUGUUUGGAGAUTT-3’
siUPP1-3	5’-CCGCUAUGCCAUGUAUAAATT-3’
**shRNA**	**Sequences**
shNC	5’-TTCTCCGAACGTGTCACGT-3’
shUPP1	5’-CCGCUAUGCCAUGUAUAAA-3’
**Overexpression lentivirus**	**Sequences**
Vector	LV5-NC (GenePharma, China) promotor: EF-1a; fluorescent label: CopGFP; eukaryotic resistance: Puro
UPP1	5’-ATGGCGGCCACGGGAGCCAATGCAGAGAAAGCTGAAAGTCACAATGATTGCCCCGTCAGACTTTTAAATCCAAACATAGCAAAAATGAAAGAAGATATTCTCTATCATTTCAATCTCACCACTAGCAGACACAATTTCCCAGCCTTGTTTGGAGATGTGAAGTTTGTGTGTGTTGGTGGAAGCCCCTCCCGGATGAAAGCCTTCATCAGGTGCGTTGGTGCAGAGCTGGGCCTTGACTGCCCAGGTAGAGACTATCCCAACATCTGTGCGGGAACTGACCGCTATGCCATGTATAAAGTAGGACCGGTGCTGTCTGTCAGTCATGGTATGGGCATTCCTTCTATCTCAATCATGTTGCATGAGCTCATAAAGCTGCTGTACTATGCCCGGTGCTCCAACGTCACTATCATCCGCATTGGCACTTCTGGTGGGATAGGTCTGGAGCCCGGCACTGTGGTCATAACAGAGCAGGCAGTGGATACCTGCTTCAAGGCAGAGTTTGAGCAGATTGTCCTGGGGAAGCGGGTCATCCGGAAAACGGACCTTAACAAGAAGCTGGTGCAGGAGCTGTTGCTGTGTTCTGCAGAGCTGAGCGAGTTCACCACAGTGGTGGGGAACACCATGTGCACCTTGGACTTCTATGAAGGGCAAGGCCGTCTGGATGGGGCTCTCTGCTCCTACACGGAGAAGGACAAGCAGGCGTATCTGGAGGCAGCCTATGCAGCCGGCGTCCGCAATATCGAGATGGAGTCCTCGGTGTTTGCCGCCATGTGCAGCGCCTGCGGCCTCCAAGCGGCCGTGGTGTGTGTCACCCTCCTGAACCGCCTGGAAGGGGACCAGATCAGCAGCCCTCGCAATGTGCTCAGCGAGTACCAGCAGAGGCCGCAGCGGCTGGTGAGCTACTTCATCAAGAAGAAACTGAGCAAGGCCTGA-3’

Supplementary Table S3. Primer sequences for UPP1-R94A construction.

Primer	Sequences
Forward Primer	5’-AACTGACGCCTATGCCATGTATAAAGTAGGACCGG-3’
Reverse Primer	5’-TGGCATAGGCGTCAGTTCCCGCACAGATGTTG-3’

Supplementary Table S4. Primer sequences for qRT-PCR.

Primer	Sequences
β-Actin-F	5’-CATGTACGTTGCTATCCAGGC-3’
β-Actin-R	5’-CTCCTTAATGTCACGCACGAT-3’
UPP1-F	5’-TGATTGCCCCGTCAGACTTTT-3’
UPP1-R	5’-CACCAACGCACCTGATGAAG-3’

Supplementary Table S5. Basic clinical information and AOD of microarrays.

Location	AOD of UPP1	Gender	Age
A01	0.267826	male	64
A02	0.091901	male	64
A03	0.31099	male	76
A04	0.167152	male	76
A05	0.219339	male	67
A06	0.087377	male	67
A07	0.259168	male	82
A08	0.171468	male	82
A09	0.268074	male	82
A10	0.27972	male	82
A11	0.24064	male	62
A12	0.27401	male	62
B01	0.365097	male	75
B02	0.134855	male	75
B03	0.2395	male	80
B04	0.168368	male	80
B05	0.271013	male	50
B06	0.271874	male	50
B07	0.263138	male	59
B08	0.303195	male	59
B09	0.281255	male	66
B10	0.245237	male	66
B11	0.166709	male	79
B12	0.034215	male	79
C01	0.174812	male	76
C02	0.231738	male	76
C03	0.228742	male	67
C04	0.269623	male	67
C05	0.292597	male	83
C06	0.165052	male	83
C07	0.262241	male	81
C08	0.145867	male	81
C09	0.256992	male	75
C10	0.245389	male	71
C11	0.255865	male	75
C12	0.255118	female	72
D01	0.260546	female	66
D02	0.240268	male	67
D03	0.24461	male	58
D04	0.250567	male	77
D05	0.236214	male	68
D06	0.260304	male	61
D07	0.245544	female	58

**Location**	**AOD of UPP1**	**Gender**	**Age**
D08	0.290709	male	73
D09	0.235669	female	42
D10	0.279172	male	57
D11	0.379501	male	55
D12	0.237131	male	75
E01	0.236565	male	73
E02	0.23893	male	77
E03	0.234914	male	57
E04	0.248062	male	78
E05	0.276594	male	74
E06	0.24586	female	72
E07	0.26937	female	65
E08	0.237768	male	59
E09	0.233318	male	75
E10	0.241617	male	55
E11	0.24965	male	57
E12	0.24531	male	61
F01	0.235941	male	79
F02	0.246294	male	77
F03	0.247821	male	48
F04	0.330338	female	x
F05	0.333878	female	85
F06	0.339982	male	76
F07	0.285269	male	61
F08	0.270913	male	75
F09	0.241989	female	66
F10	0.235141	male	84
F11	0.248011	male	75
F12	0.236768	male	78
G01	0.206842	male	44
G02	0.210028	female	77
G03	0.24098	male	71
G04	0.377985	male	62
G05	0.255086	male	59
G06	0.238955	male	64
G07	0.240774	male	67
